# Machine‐Learning‐Guided Discovery of Boron‐Free Narrowband Blue Thermally Activated Delayed Fluorescence Emitters

**DOI:** 10.1002/anie.2731055

**Published:** 2026-07-21

**Authors:** Masaya Hagai, Jie Sun, Jun Hyeon Lee, Kazuhiro J. Fujimoto, Shigehiro Yamaguchi, Takuma Yasuda, Takeshi Yanai

**Affiliations:** ^1^ Department of Chemistry Graduate School of Science Nagoya University Nagoya Japan; ^2^ Department of Applied Chemistry Graduate School of Engineering Kyushu University Fukuoka Japan; ^3^ Institute of Transformative Bio‐Molecules (WPI‐ITbM) Nagoya University Nagoya Japan; ^4^ Institute for Advanced Study Kyushu University Fukuoka Japan

**Keywords:** graph neural network, high‐throughput screening, organic light‐emitting diodes, polycyclic aromatic hydrocarbons, thermally activated delayed fluorescence

## Abstract

Thermally activated delayed fluorescence (TADF) emitters enable color‐pure, energy‐efficient organic light‐emitting diodes (OLEDs), yet many ultra‐narrowband designs rely on synthetically demanding boron incorporation. Here we discover boron‐free emitters that reconcile a robust nitrogen‐only polycyclic scaffold, Rec. 2020‐grade pure‐blue emission, and small singlet–triplet gaps (Δ*E*_ST_) conducive to TADF. We enumerated 19 518 13‐ring carbazole‐containing polycyclic aromatic hydrocarbons (Cz‐PAHs) and obtained quantum‐chemical reference values for ∼1000 molecules: emission energies and Δ*E*_ST_ at S_1_‐optimized geometries. A graph neural network enabled library‐wide prediction and revealed a pronounced negative correlation between emission energy and Δ*E*_ST_ (*R* = −0.75), implicating configuration mixing in S_1_ and indicating that small‐gap candidates are enriched in the pure‐blue regime. Two selected Cz‐PAH emitters were synthesized and display deep‐blue, exceptionally narrow photoluminescence (full widths at half maximum 17–19 nm) with small experimental Δ*E*_ST_ (0.13–0.19 eV). OLEDs incorporating these emitters deliver narrowband blue electroluminescence (*λ*_EL_ 456 and 481 nm); device A reaches chromaticity coordinates of (0.139, 0.069), close to the Rec. 2020 blue primary, whereas the sensitized device D achieves a maximum external quantum efficiency of 35.2%. Collectively, this work expands the boron‐free TADF design space and establishes an experimentally validated route to discovering high‐color‐purity organic emitters.

## Graphical abstract

A data‐driven discovery engine—exhaustive enumeration of carbazole‐containing polycyclic aromatic hydrocarbons (Cz‐PAHs) followed by machine learning—identifies boron‐free, narrowband blue thermally activated delayed fluorescence emitters for organic light‐emitting diodes (OLEDs). The synthesized lead emitter delivers intense deep‐blue photoluminescence (PL) and device chromaticity close to the Rec. 2020 blue primary.

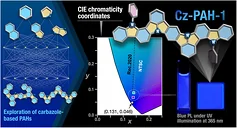

## 1 Introduction

Multi‐resonance thermally activated delayed fluorescence (MR‐TADF) [1] emitters have emerged as a powerful molecular paradigm for organic light‐emitting diodes (OLEDs), enabling noble‐metal‐free, near‐unity internal quantum efficiency together with the intrinsically narrow emission bandwidths required to meet Rec. 2020 (BT.2020) color standards for ultra‐high‐definition displays [2, 3, 4, 5]. In TADF systems, non‐emissive triplet (T_1_) excitons are thermally upconverted to emissive singlet (S_1_) excitons via reverse intersystem crossing (RISC), a process governed by the singlet–triplet energy gap (Δ*E*_ST_) and spin–orbit coupling (SOC) [6, 7, 8]. Although conventional twisted donor–acceptor TADF architectures [6, 7, 8] can achieve small Δ*E*_ST_, their strong intramolecular charge‐transfer character induces substantial excited‐state relaxation, resulting in broad photoluminescence (PL) and electroluminescence (EL) spectra and compromised color purity [2]. By contrast, prototypical MR‐TADF emitters are built on rigid, heteroatom‐doped polycyclic aromatic hydrocarbons (PAHs), in which electron‐donating and electron‐accepting atoms (e.g., N and B) are arranged in an opposing resonance pattern. This atomic‐scale segregation of frontier molecular orbitals suppresses excited‐state reorganization and vibronic coupling, affording narrowband emission with full width at half maximum (FWHM) <30 nm [1] while maintaining high PL quantum yield (*Φ*_PL_) and small Δ*E*_ST_. These features underpin the exceptional color fidelity achieved in MR‐TADF‐based OLEDs.

Alongside B/N‐doped MR systems, boron‐free narrowband TADF emitters based on carbazole (Cz)‐embedded PAH frameworks (here denoted Cz‐PAHs) have attracted increasing attention [9, 10, 11, 12, 13, 14, 15, 16, 17, 18, 19, 20]. Composed solely of C, H, and N atoms, Cz‐PAHs preserve MR‐like frontier‐orbital alternation with short‐range charge transfer, thereby supporting small Δ*E*_ST_ without sacrificing oscillator strength. Importantly, their synthetic accessibility—through established C–C and C–N cross‐coupling reactions combined with oxidative cyclodehydrogenation—allows efficient modular diversification [21, 22]. The intrinsic rigidity of Cz‐PAH scaffolds suppresses geometric relaxation and vibronic progression, yielding narrow emission bandwidths with well‐defined Commission Internationale de l'Éclairage (CIE) chromaticity coordinates. State‐of‐the‐art Cz‐PAHs have achieved spectral FWHMs approaching ∼20 nm together with high *Φ*_PL_ and small Δ*E*_ST_.

In TADF OLEDs, the device‐relevant figure of merit is the reverse intersystem crossing rate, *k*_RISC_. While a small Δ*E*_ST_ is generally favorable, *k*_RISC_ is governed by a concert of effects—nonadiabatic spin–vibronic coupling (NA‐SVC) among S_1_ and nearby T_n_ states and vibrationally induced SOC (Herzberg–Teller (HT)‐SVC). As a result, reliable prediction typically requires excited‐state treatments beyond Marcus‐theory‐based rate models relying primarily on vertical energy gaps and direct (Condon) SOC. A unified framework incorporating both NA‐SVC and HT‐SVC (the 2nd+HT model) reproduces MR‐TADF trends with significantly higher accuracy than simplified schemes [23, 24]. However, the associated computational cost precludes library‐scale studies; accordingly, high‐throughput screening commonly adopts Δ*E*_ST_ as an operational surrogate for *k*_RISC_.

This reliance exposes a central bottleneck in rational discovery: obtaining decision‐grade Δ*E*_ST_ across expansive libraries rapidly enough to guide design and prioritization. Achieving such accuracy typically demands high‐level quantum‐chemical methods (e.g., equation‐of‐motion coupled‐cluster singles and doubles at the second‐order approximation (EOM‐CC2) [25] and spin‐component‐scaled CC2 (SCS‐CC2) [26]). While pioneering studies have demonstrated large‐scale virtual screening of excited‐state properties using such approaches [27], the required computational resources remain substantial, limiting routine exhaustive exploration. Conversely, machine‐learning (ML) models offer high throughput [28, 29, 30, 31, 32, 33, 34, 35], but their precision and robustness outside the training domain remain inadequate for confident prioritization. The resulting accuracy–throughput trade‐off continues to impede accelerated materials discovery in this materials space.

Systematic enumeration of constrained aromatic design spaces offers a practical route to mitigate the accuracy–throughput bottleneck in excited‐state screening. Representative platform efforts include COMPAS, which assembles large‐scale libraries of condensed (hetero)aromatic systems—up to ∼5 × 10^5^ molecules—with optimized geometries and computed electronic properties to enable data‐driven exploration [36, 37, 38]. Complementing these resources, our prior exhaustive generator produced ∼2,500 dithieno‐diboraperylene variants for time‐dependent density functional theory (TDDFT) [39] screening, yielding a near‐infrared‐emissive candidate that was subsequently synthesized and validated [40]. These studies demonstrate that enumeration can reveal structure–property trends and surface unexpected candidates. However, fully enumerative workflows are computationally demanding and scale poorly beyond narrowly defined scaffold families. This limitation becomes particularly acute for MR‐TADF optimization, where reliable candidate selection depends on accurate excited‐state descriptors such as Δ*E*_ST_, rendering exhaustive exploration of the expansive design space impractical.

Boron‐free Cz‐PAH systems can deliver narrowband emission, yet many reported structures retain comparatively large Δ*E*_ST_ (> 0.3 eV), which suppresses RISC and limits TADF efficiency. Simultaneously achieving a small Δ*E*_ST_ and narrow emission bandwidth therefore requires identifying new core topologies across an expansive PAH design space—beyond the practical reach of empirical iteration or exhaustive quantum‐chemical screening. An ML‐guided, high‐throughput discovery framework tailored to Cz‐PAH scaffolds is therefore needed, preserving MR‐like orbital localization while directly optimizing Δ*E*_ST_.

In defining the target chemical space, we selected 13‐ring‐fused Cz‐PAHs as a chemically focused compromise between photophysical performance and experimental tractability. Smaller fused frameworks tend to have wider optical gaps, which can shift emission toward the near‐ultraviolet, whereas excessive π‐extension often introduces solubility, sublimation, and processing penalties. The 13‐ring framework is therefore sufficiently extended and rigid to support deep‐blue, narrowband emission while remaining compatible with synthesis and OLED fabrication. This choice is also consistent with recent demonstrations that rigid heteroaromatic frameworks and extended multiple‐resonance effects are key to achieving high efficiency and superior color purity [41, 42].

Guided by this design rationale, we here present an ML‐guided discovery workflow for boron‐free, narrowband blue TADF emitters that integrates exhaustive enumeration of 13‐ring‐fused Cz‐PAHs with a graph neural network (GNN) trained on high‐level excited‐state calculations. A randomly sampled subset of the enumerated library is computed at the TDDFT and SCS‐CC2 levels of theory to obtain quantum‐chemical reference values—fluorescence emission energy (*λ*_em_) and Δ*E*_ST_—which serve as training targets for the GNN; the trained model then predicts these quantities across the full library, enabling rapid candidate triage and prioritization. Structures satisfying predefined photophysical criteria and synthetic feasibility constraints are synthesized and experimentally characterized as OLED emitters. By confining the search to a chemically well‐defined design space while combining quantum‐chemical labels with scalable ML inference, this strategy achieves accuracy at scale and establishes a generalizable framework for the property‐driven discovery of narrowband TADF emitters.

## 2 Results and Discussion

### 2.1 Exhaustive Enumeration of 13‐Ring‐Fused Cz‐PAHs

**FIGURE 1:**
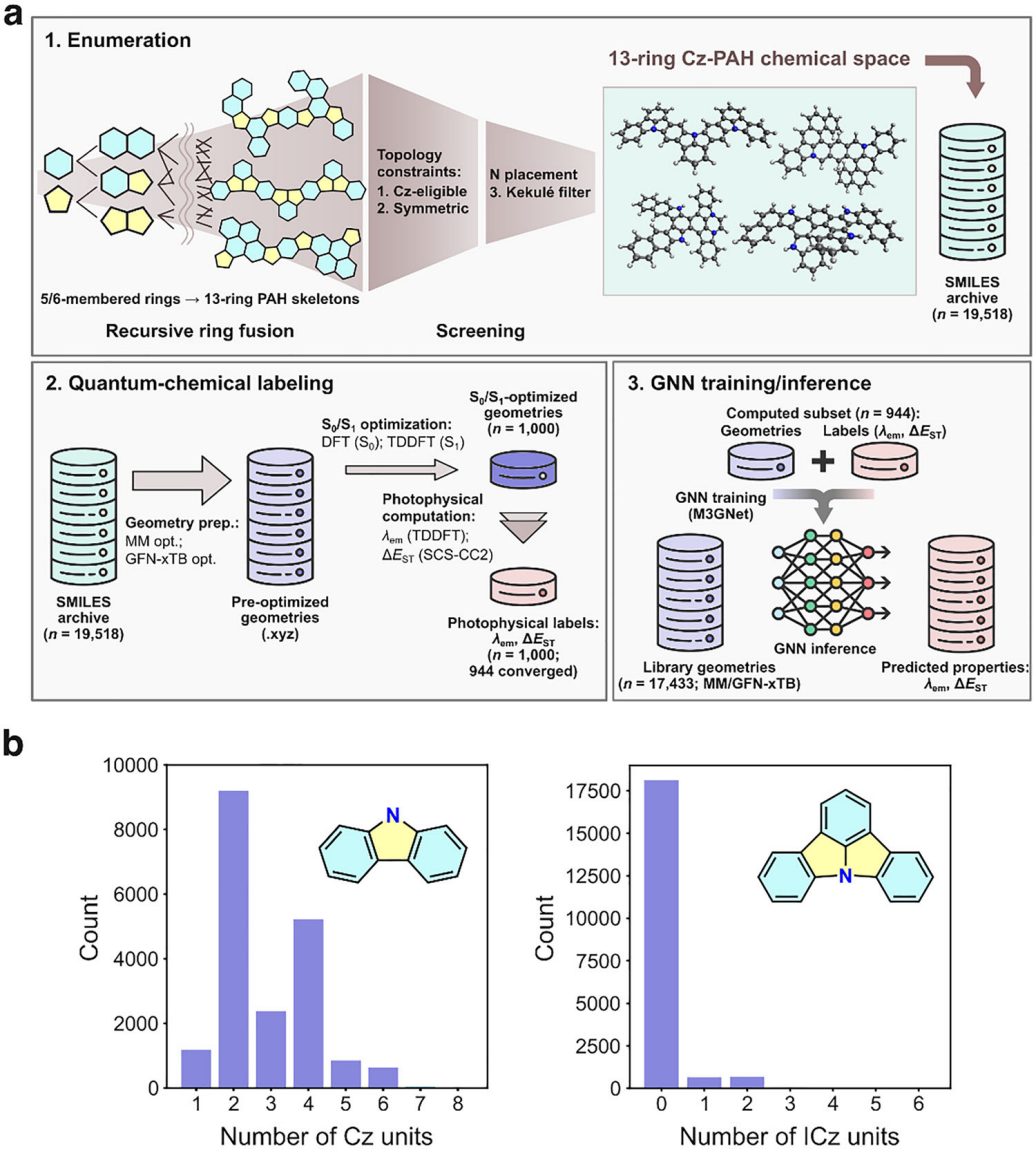
Workflow for discovery of boron‐free TADF emitters and overview of the Cz‐PAH design space. (a) (1) Enumeration. Recursive 5/6‐ring fusion yields 13‐ring PAH skeletons; Cz‐eligible and symmetric scaffolds are retained; nitrogen is introduced; only Kekulé‐assignable scaffolds are kept, producing a SMILES archive (*n* = 19 518). (2) Quantum‐chemical labeling. From SMILES, geometries are prepared and optimized; photophysical labels—fluorescence energy (*λ*_em_, TDDFT) and singlet–triplet gap (Δ*E*_ST_, SCS‐CC2)—are computed at S_1_‐optimized geometries, yielding the reference dataset (*n* = 1000; 944 converged). (3) GNN training/inference. The converged subset (*n* = 944) with geometries and labels is used to train the GNN (M3GNet); the trained model predicts *λ*_em_ and Δ*E*_ST_ for the remaining 17 433 structures. (b) Substructure statistics. Distributions of carbazole (Cz) and indolo[3,2,1‐*jk*]carbazole (ICz) counts per molecule across the enumerated library.

As outlined in Figure 1a, the workflow proceeds from exhaustive enumeration (this section) to quantum‐chemical labeling and then to GNN training/inference. An in‐house molecular generator [40] was employed to exhaustively construct PAH skeletons and corresponding heteroatom‐embedded derivatives. Applied to 13‐ring‐fused topologies, the generator systematically enumerated all PAH skeletons accessible by recursive fusion of five‐ and/or six‐membered rings, yielding a chemically diverse yet topologically well‐defined design space. To ensure incorporation of at least one Cz motif, the enumeration was restricted to scaffolds in which every five‐membered ring is annulated on both sides by six‐membered rings. Synthetic tractability was further promoted by prioritizing symmetric frameworks. Nitrogen atoms were then introduced at positions that generate Cz substructures, and only molecules admitting a valid Kekulé representation were retained. This workflow yielded a library of 19 518 unique Cz‐PAHs, which were exported as SMILES strings and curated in a dedicated database (Figure 1a).

The resulting library spans a remarkably broad range of Cz‐embedding patterns and local π‐conjugation topologies, highlighting the diversity of accessible chemical space. Substructure analysis (Figure 1b) revealed that approximately half of the library (9206 of 19 518 structures) contains two Cz subunits, whereas a small subset incorporates seven or more and was identified as sterically congested and non‐planar. Indolo[3,2,1‐*jk*]carbazole (ICz) motifs are notably rare, with 93% of the library lacking this substructure altogether; where present (up to six per molecule), the geometries were typically sterically unstable.

This low ICz fraction reflects the unbiased enumeration strategy rather than an exclusion of ICz‐based motifs. ICz units were allowed to emerge as specific substructures within the broader Cz‐PAH design space, but were not explicitly enforced or enriched during molecular generation. This choice avoids biasing the library toward already established ICz‐based design motifs, despite their demonstrated success in high‐performance blue TADF emitters [10, 13, 14, 15, 43], and instead enables discovery across a broader topological space.

### 2.2 Quantum‐Chemical Labeling

From the enumerated library of 19 518 Cz‐PAHs, 1000 molecules were randomly sampled for high‐accuracy excited‐state calculations. Fluorescence emission energies (*λ*_em_, used here as a proxy for emission color) were computed with TDDFT [39], and Δ*E*_ST_, a key descriptor for RISC, with SCS‐CC2 [26]. Of these, 944 calculations converged successfully and were retained for subsequent analysis (Figure 1a).

**FIGURE 2:**
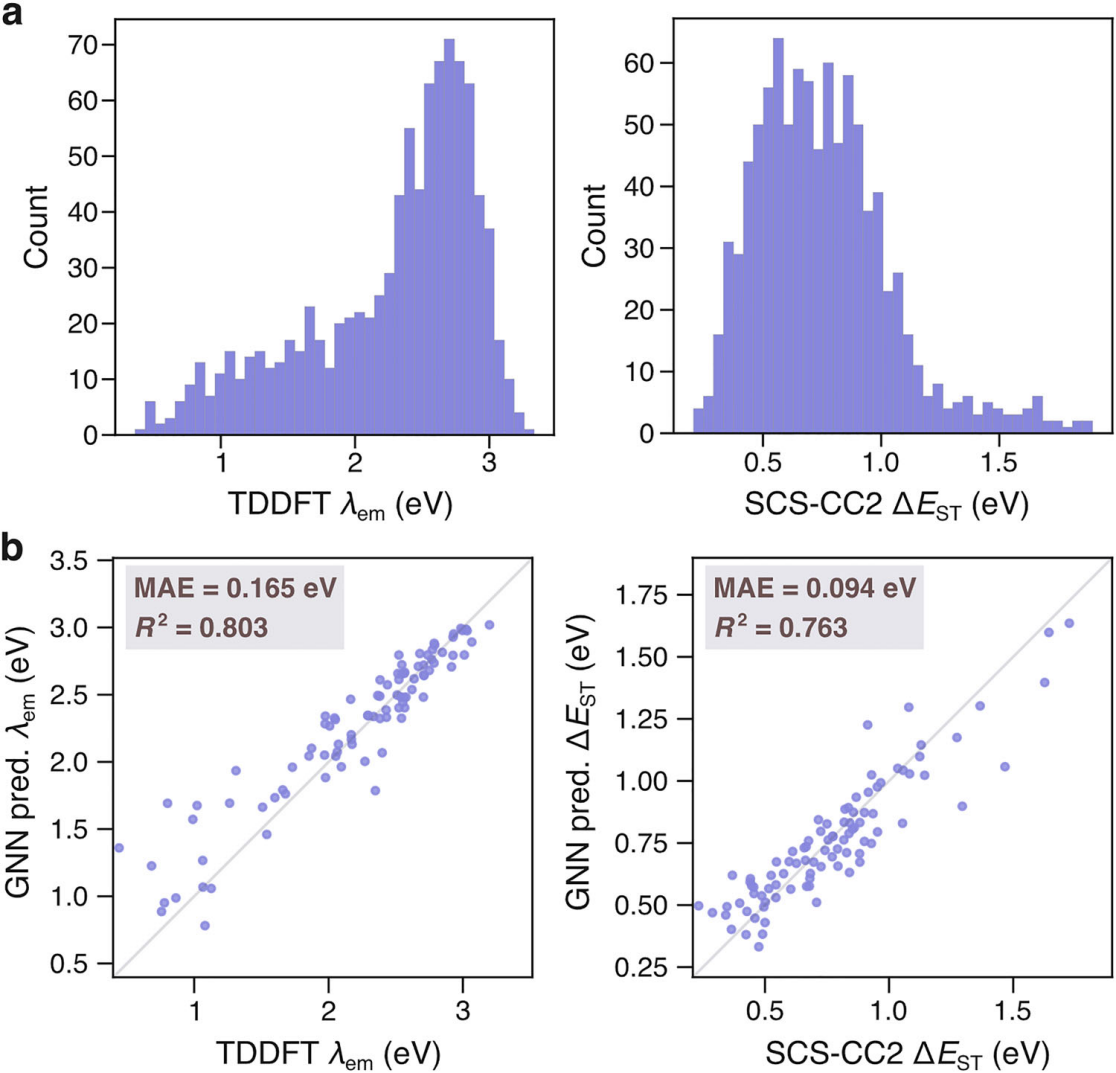
Reference property distributions and GNN predictive performance. (a) TDDFT *λ*_em_ and SCS‐CC2 Δ*E*_ST_ distributions for the computed subset (*n* = 944). *λ*_em_ is unimodal (mean 2.27 eV, SD 0.64 eV; range 0.36–3.33 eV). Δ*E*_ST_ is approximately Gaussian (mean 0.75 eV, SD 0.29 eV; range 0.21–1.89 eV); 13 molecules (1.4%) satisfy Δ*E*_ST_ < 0.30 eV. (b) GNN predictions versus quantum‐chemical references for *λ*_em_ (left) and Δ*E*_ST_ (right) on the test set. The solid line denotes *y* = *x*; *R* ^2^ = 0.80 for *λ*_em_ and *R* ^2^ = 0.76 for Δ*E*_ST_.

The TDDFT‐computed *λ*_em_ values exhibit a unimodal distribution (Figure 2a) with mean 2.27 eV and standard deviation (SD) 0.64 eV, centered in the violet–green spectral region. Approximately 28% of molecules display *λ*_em_ values below 2.0 eV, corresponding to red‐emissive candidates. Overall, *λ*_em_ spans 0.36–3.33 eV (3444–372 nm), demonstrating substantial spectral diversity—from the mid‐infrared to the near‐ultraviolet—within a single Cz‐PAH structural family.

The SCS‐CC2‐computed Δ*E*_ST_ values follow an approximately Gaussian distribution (Figure 2a), with mean 0.75 eV and SD 0.29 eV, spanning 0.21–1.89 eV. Notably, only 13 molecules (1.4% of the dataset) exhibit Δ*E*_ST_ values below 0.3 eV—a threshold generally considered favorable for efficient TADF.

### 2.3 GNN Training and Inference

A GNN (M3GNet [44]) was trained to capture structure–property relationships from the quantum‐chemical dataset (Figure 1a). SMILES‐derived geometries were first preoptimized by molecular mechanics (MM) and then optimized at the GFN‐xTB level. The resulting GFN‐xTB geometries were used as structural inputs for both GNN training and library‐scale inference, whereas the target properties were evaluated from the corresponding higher‐level S_1_‐optimized geometries. Thus, the model learned to associate inexpensive xTB‐level input structures with higher‐level excited‐state properties.

The 944 molecules with fully converged quantum‐chemical calculations were randomly split into training, validation, and test sets in an 8:1:1 ratio. On the held‐out test set, the model showed strong predictive performance, achieving coefficients of determination of *R* ^2^ = 0.80 for *λ*_em_ and *R* ^2^ = 0.76 for Δ*E*_ST_ (parity plots in Figure 2b). Prediction errors for *λ*_em_ were moderately larger in the low‐energy region below 2.0 eV, likely reflecting known TDDFT limitations and the smaller number of low‐energy emitters in the training set. Likewise, for Δ*E*_ST_, the limited number of training samples above 1.0 eV resulted in slightly larger prediction errors in that range.

The main calibration limitation occurs at the lower tail of the Δ*E*_ST_ distribution. Because only 13 of the 944 reference molecules (1.4%) had Δ*E*_ST_ < 0.3 eV, the GNN tends to overestimate exceptionally small gaps by regressing toward the more densely populated region of the dataset. Accordingly, the model was used primarily as a library‐scale prescreening and ranking tool, whereas final candidate prioritization relied on subsequent higher‐level SCS‐CC2 and TDDFT calculations.

**FIGURE 3:**
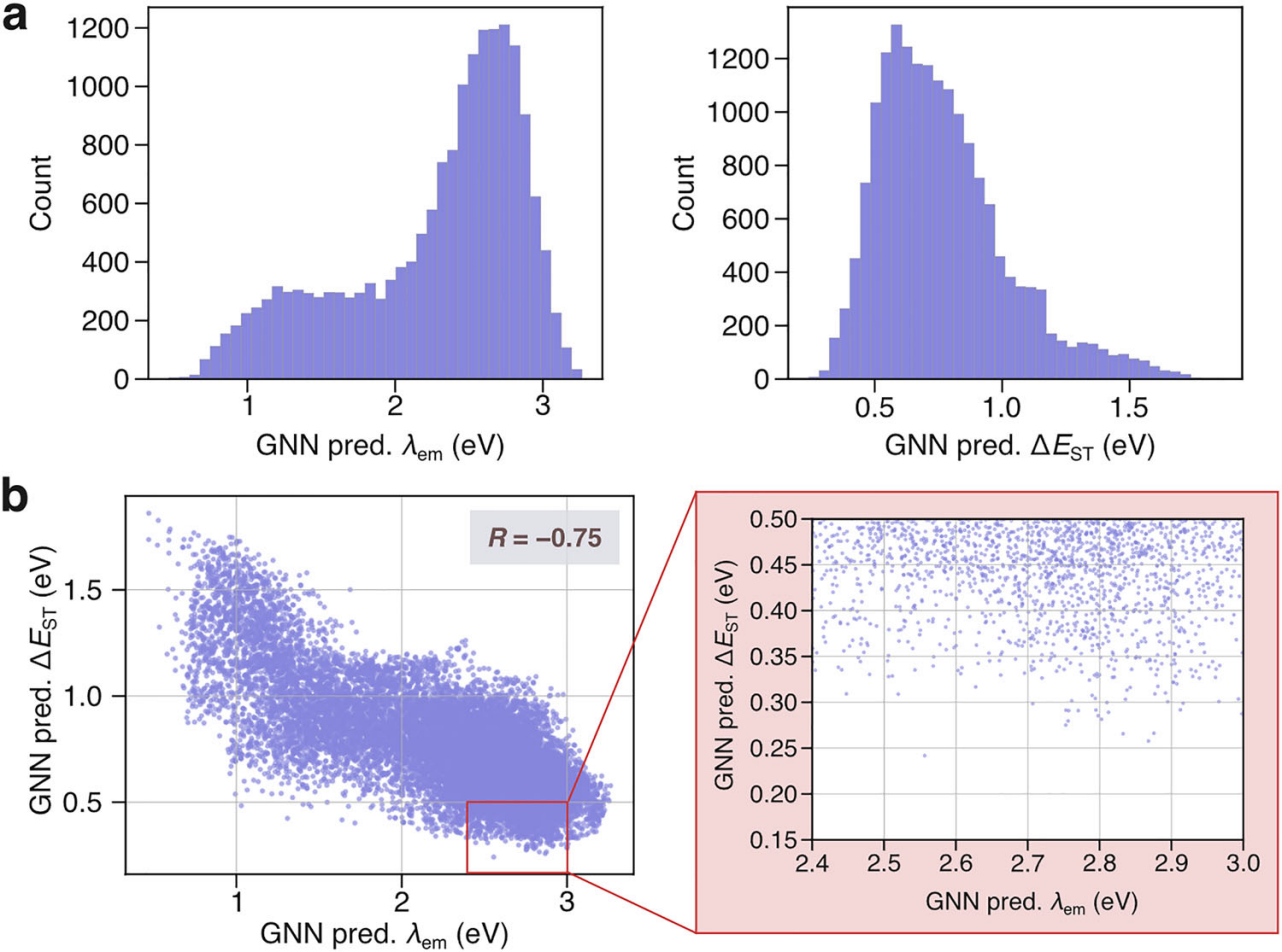
Library‐wide GNN inference and design‐relevant trends. (a) GNN‐predicted *λ*_em_ (left) and Δ*E*_ST_ (right) across the remaining 17 433 13‐ring Cz‐PAHs; distributions closely match the quantum‐chemically computed subset (*n* = 944). (b) GNN‐predicted *λ*_em_ versus Δ*E*_ST_ across the full library, showing a negative correlation (Pearson *R* = −0.75). Right panel: blue‐emission window (restricted to Δ*E*_ST_ < 0.5 eV and *λ*_em_ = 2.4–3.0 eV), where smaller‐Δ*E*_ST_ candidates are concentrated at higher *λ*_em_ (bluer side).

Using the same input protocol, the trained GNN model was then applied to infer *λ*_em_ and Δ*E*_ST_ across the full 13‐ring‐fused Cz‐PAH library (Figure 1a). Valid MM/GFN‐xTB‐optimized geometries were obtained for 17 433 of the 18 518 SMILES entries, and predictions were performed for this subset. The predicted histograms (Figure 3a) closely matched those obtained for the quantum‐chemically computed subset (Figure 2a), demonstrating robust generalization across the expanded chemical space. Notably, the mean predicted *λ*_em_ (2.27 eV) was nearly identical to that of the computed subset (2.27 eV), and the mean predicted Δ*E*_ST_ likewise showed close agreement (0.77 eV vs. 0.75 eV). This agreement supports our GNN as a reliable surrogate for high‐throughput screening of emission and excited‐state properties beyond the sampled subset.

Across the full Cz‐PAH library, the GNN‐predicted *λ*_em_ and Δ*E*_ST_ exhibit a pronounced negative correlation (Pearson *R* = −0.75; Figure 3b). In a single‐excitation (HOMO–LUMO) approximation, both quantities would be expected to increase together with the HOMO–LUMO exchange interaction (*K*_HOMO, LUMO_) [45, 46]. The observed inverse trend is therefore inconsistent with a purely HOMO–LUMO description and instead implies substantial configuration mixing in S_1_, with contributions from deeper occupied and/or higher‐lying virtual orbitals beyond a single HOMO–LUMO transition. This observation points to design strategies that go beyond tuning only frontier orbitals and instead aim to control configuration mixing and orbital interactions that govern both emission energy and RISC efficiency.

When restricted to emitters with Δ*E*_ST_ < 0.5 eV and *λ*_em_ of 2.4–3.0 eV, candidates with smaller Δ*E*_ST_ tend to fall toward the higher‐energy (bluer) end of the distribution (Figure 3b). In contrast, previously reported green‐emitting Cz‐PAHs generally exhibit larger Δ*E*_ST_ values [47, 48, 49], thereby underscoring a practical design principle for efficient blue TADF emitters: within the relevant spectral window, candidates that sustain small Δ*E*_ST_ should be prioritized.

Guided by the GNN predictions, a hierarchical screening workflow was implemented. Candidates were first filtered to those with predicted *λ*_em_ within the pure‐blue window (2.4–3.0 eV), consistent with the requirements for high‐color‐purity display applications. The initial screening criteria were intentionally conservative in light of the GNN prediction errors (MAEs of 0.165 eV for *λ*_em_ and 0.094 eV for Δ*E*_ST_), prioritizing the avoidance of false negatives over immediate quantitative precision. From this broadly defined candidate set, the 50 molecules with the smallest predicted Δ*E*_ST_ (Figure S1) were subjected to higher‐level SCS‐CC2 calculations to rigorously refine Δ*E*_ST_ and prioritize structures with favorable TADF potential, thereby narrowing the pool to five leading candidates. Further consideration of synthetic accessibility and structural feasibility ultimately identified two targets, Candidates 02 and 05 (Figure S1), which were advanced to experimental verification.

Consistent with the lower‐tail calibration bias discussed above, the GNN‐predicted Δ*E*_ST_ values for Candidates 02 and 05 (0.33 and 0.28 eV, respectively) were higher than the subsequent SCS‐CC2 values (Table S1). Nevertheless, both molecules were retained within the initial top‐50 pool, corresponding to the top ∼0.3% of the generated library. This outcome supports the intended use of the GNN as a library‐scale prescreening and ranking tool, with final prioritization based on higher‐level quantum‐chemical evaluation.

The structures of the two selected emitters also provide a useful retrospective validation of the unbiased enumeration strategy. Although ICz motifs were not explicitly enriched during molecular generation and account for only ∼7% of the library, both Candidates 02 and 05 contain bis‐ICz cores. Thus, the ML‐guided workflow recovered favorable ICz‐based architectures directly from the broader Cz‐PAH design space, consistent with prior evidence that fused ICz motifs can promote small Δ*E*_ST_ and efficient blue TADF behavior.

### 2.4 Experimental Validation of Narrowband Blue Luminescence

**FIGURE 4:**
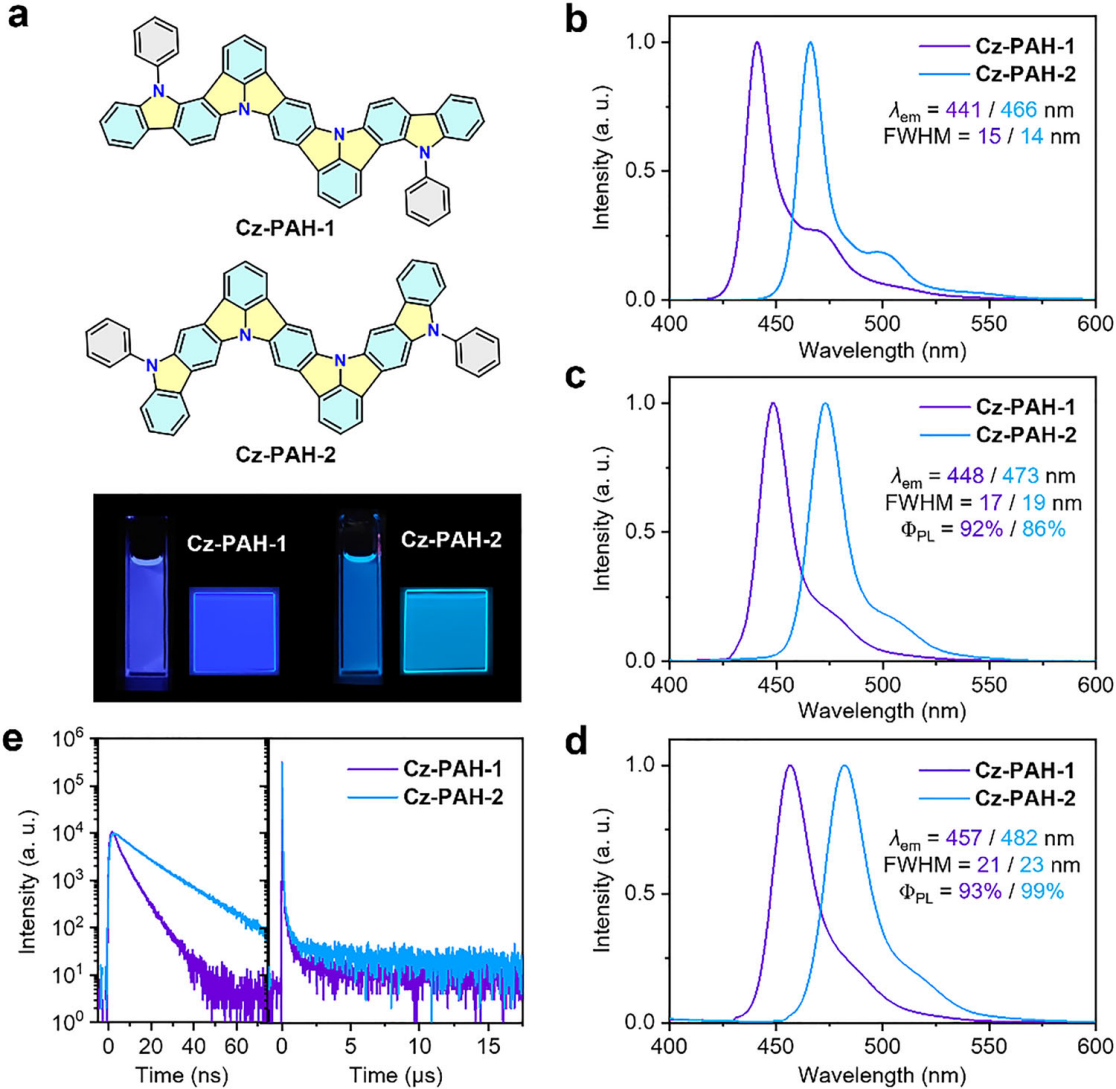
Photophysical characterization of **Cz‐PAH‐1** and **Cz‐PAH‐2**. (a) Molecular structures of the two experimentally validated emitters and photographs showing blue PL under UV illumination at 365 nm. (b) Simulated emission spectra computed using an in‐house implementation [23, 50] of the thermal vibration correlation function (TVCF) [51] method based on TDDFT calculations. (c) Experimental emission spectra measured in toluene solution (10^−5^ M). (d) Experimental emission spectra measured for Cz‐PAH:mCPBC (1:99, w/w)‐doped films. (e) Transient PL decay curves in the nanosecond and microsecond regimes for the Cz‐PAH:mCPBC (1:99, w/w)‐doped films.

**FIGURE 5:**
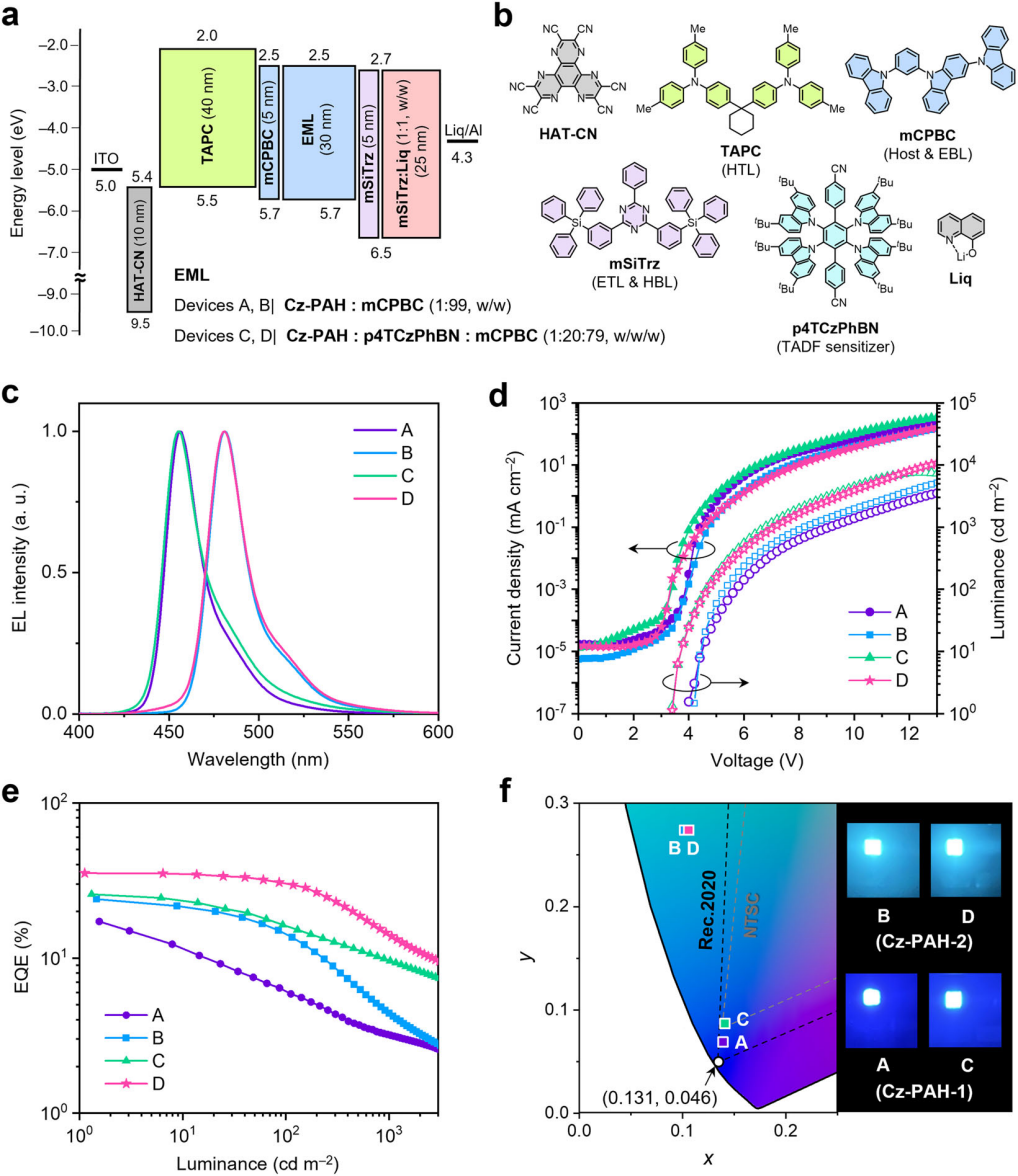
EL performance of devices A–D. (a) OLED device architecture with energy‐level diagram. (b) Chemical structures of materials used in the OLEDs. (c) EL spectra measured at 1 mA cm^−2^. (d) Current density and luminance versus voltage (*J*–*V*–*L*) characteristics. (e) External quantum efficiency versus luminance (EQE–*L*) characteristics. (f) EL emission images and the corresponding CIE chromaticity coordinates.

For experimental validation, Candidates 02 and 05 were synthesized as the corresponding N‐phenyl (N–Ph)‐capped derivatives, designated **Cz‐PAH‐1** and **Cz‐PAH‐2**, respectively (Figure 4a). The N–Ph capping was introduced to improve molecular stability and processability for OLED fabrication while preserving the electronic characteristics of the parent Cz‐PAH frameworks. Relative to the calculated properties of the parent cores (Table S1), the N–Ph‐capped derivatives showed only marginal changes in the targeted photophysical descriptors, retaining blue emission energies (*λ*_em_ = 2.83 and 2.68 eV), small reorganization energies (*Λ* = 0.13 and 0.10 eV), and small Δ*E*_ST_ values (0.12 and 0.16 eV) for **Cz‐PAH‐1** and **Cz‐PAH‐2**, respectively. This constellation of properties is expected to suppress excited‐state structural relaxation while maintaining thermally accessible RISC, rendering these molecules promising narrowband blue TADF candidates. Both compounds were synthesized from boronic acid‐substituted 9‐phenylcarbazoles via a convergent five‐step sequence comprising Suzuki–Miyaura coupling, Cadogan cyclization, Miyaura–Ishiyama borylation, a second Suzuki–Miyaura coupling, and intramolecular Ullmann C–N coupling (see Supporting Information for details). Thermogravimetric analysis confirmed the excellent thermal robustness of both emitters, with 5% weight‐loss decomposition temperatures (*T*_d_) of 446°C for **Cz‐PAH‐1** and 472°C for **Cz‐PAH‐2** (**Figure** **S7**). These high *T*_d_ values support their compatibility with vacuum processing. We then examined their photophysical properties in solution and in doped thin films (Figure 4, Tables S4 and S5) and subsequently evaluated their EL performance in OLED devices (Figure 5).

In dilute toluene solution, **Cz‐PAH‐1** and **Cz‐PAH‐2** exhibited intense blue PL with emission maxima at *λ*_em_ = 448 and 473 nm (2.77 and 2.62 eV), respectively, closely matching the simulated spectra in both emission energy and overall spectral lineshape (Figure 4b,c). Notably, both emitters displayed exceptionally narrow emission bandwidths, with FWHMs of only 17 and 19 nm, respectively. In addition, Δ*E*_ST_ values as small as 0.13 and 0.19 eV were determined for **Cz‐PAH‐1** and **Cz‐PAH‐2**, respectively (Figure S8), corroborating their intrinsic TADF capability. Because the SCS‐CC2 calculations were performed for isolated molecules, these solution‐state Δ*E*_ST_ values serve as the most appropriate experimental benchmarks for comparison with the calculated Δ*E*_ST_ values.

When doped into 9‐(3‐(9*H*‐carbazol‐9‐yl)phenyl)‐9*H*‐3,9'‐bicarbazole (mCPBC) host films at a concentration of 1 wt%, both emitters retained their narrowband emission characteristics, showing slight bathochromic shifts in *λ*_em_ relative to solution (Figure 4d). High *Φ*_PL_ values (93% and 99%) were achieved in the doped films, indicative of efficient radiative decay and suppressed nonradiative losses. Time‐resolved PL measurements revealed prompt and delayed emission components (Figure 4e), confirming TADF behavior, with delayed emission lifetimes of 22 µs (**Cz‐PAH‐1**) and 15 µs (**Cz‐PAH‐2**). Overall, the measured *λ*_em_, spectral bandwidths (FWHM), and Δ*E*_ST_ agreed closely with the GNN predictions. These results provide compelling validation of the GNN‐guided identification of narrowband blue TADF emitters and underscore the power of data‐driven screening in bridging in silico design and experimental realization.

### 2.5 OLED Device Performance

Encouraged by the favorable photophysical properties of **Cz‐PAH‐1** and **Cz‐PAH‐2**, OLEDs were fabricated to evaluate their performance under electrical excitation (Figure 5 and Table 1). Both emitters were first examined as standalone TADF emitters in devices A and B, employing emission layers (EMLs) consisting of Cz‐PAH doped into mCPBC, mirroring the conditions used for the photophysical characterization. Devices A and B exhibited efficient, narrowband blue EL, with emission maxima (*λ*_EL_) of 456 and 481 nm and maximum external quantum efficiencies (EQE_max_) of 17.2% and 24.0%, respectively, confirming efficient harvesting of electrically generated triplet excitons via TADF within the Cz‐PAH emitters. The corresponding CIE coordinates were (0.139, 0.069) for device A (**Cz‐PAH‐1**) and (0.102, 0.274) for device B (**Cz‐PAH‐2**). Notably, the EL of device A closely approaches the Rec. 2020 blue primary (0.131, 0.046) (Figure 5f), underscoring the capacity of these boron‐free emitters to achieve the ultra‐high color purity demanded by next‐generation display technologies.

**TABLE 1:** Summary of EL performance metrics for Cz‐PAH‐based blue OLEDs.

Device	λ_EL_ ^a^ (nm)	FWHM^b^ (nm)	CIE^c^ (*x*, *y*)	EQE_max_ ^d^ (%)	EQE_100/1000_ ^e^ (%)	CE^f^ (cd A^−1^)	PE^g^ (lm W^−1^)
A	456	22	(0.139, 0.069)	17.2	5.9/3.2	13	10
B	481	24	(0.102, 0.274)	24.0	13.6/4.4	39	27
C	455	24	(0.141, 0.087)	25.7	16.0/9.6	23	18
D	481	25	(0.106, 0.274)	35.2	30.0/14.0	52	41

^a^ EL emission maximum at 1 mA cm^−2^.

^b^ Full width at half maximum of the EL spectrum.

^c^ CIE chromaticity coordinates.

^d^ Maximum external EL quantum efficiency.

^e^ External EL quantum efficiencies at luminances of 100 and 1000 cd m^−2^.

^f^ Maximum current efficiency.

^g^ Maximum power efficiency.

To further exploit their intrinsically narrow emission profiles while maximizing EL efficiency, **Cz‐PAH‐1** and **Cz‐PAH‐2** were subsequently employed as terminal dopants in hyperfluorescence [52] (HF) OLED architectures (devices C and D), using p4TCzPhBN [53] as a TADF sensitizer. In these systems, excitons generated on the TADF sensitizer are transferred to the terminal dopants via Förster resonance energy transfer (FRET), enabling highly efficient exciton utilization while preserving the narrowband emission features of **Cz‐PAH‐1** and **Cz‐PAH‐2**. This transfer process is supported by pronounced spectral overlap between the PL spectrum of p4TCzPhBN and the absorption spectra of **Cz‐PAH‐1** and **Cz‐PAH‐2** (Figure S9). Quantitative FRET analysis gave Förster radii of 3.2 and 3.0 nm, respectively, both exceeding the estimated average donor–acceptor distance of 2.4 nm, together with FRET rate constants of ≈10^9^ s^−1^ (Table S6).

Consistent with these results, the HF devices C and D exhibited terminal‐dopant‐dominated EL spectra and substantially enhanced EL performance relative to the non‐sensitized devices A and B, delivering markedly improved EQE_max_ values of 25.7% and 35.2%, respectively. Moreover, the efficiency roll‐off was substantially suppressed at high luminance, indicative of efficient exciton management via TADF‐mediated sensitization.

Collectively, these results demonstrate that **Cz‐PAH‐1** and **Cz‐PAH‐2** operate effectively both as standalone narrowband TADF emitters and as high‐performance HF terminal dopants in OLEDs. The excellent device performance achieved by materials identified through ML‐guided prediction of fundamental photophysical parameters validates the practical utility of the GNN‐driven discovery strategy. More broadly, this work highlights data‐driven screening as a powerful platform for the rational development of high‐efficiency, color‐pure blue OLED emitters.

## 3 Conclusion

We report an ML‐guided, high‐throughput discovery framework for an advanced class of light‐emitting materials that integrates three essential attributes: a robust boron‐free scaffold, narrowband pure‐blue emission, and a small Δ*E*_ST_ enabling TADF. Leveraging a GNN surrogate model trained on an ≈1000‐molecule quantum‐chemical reference set, exhaustive generation of 13‐ring‐fused Cz‐PAH skeletons (19 518 members) was translated into accurate, library‐wide predictions of *λ*_em_ and Δ*E*_ST_ and decisive prioritization of synthetic targets. OLEDs built from the top candidates exhibit narrowband blue EL with CIE coordinates of (0.139, 0.069) and (0.102, 0.274); notably, the former lies close to the Rec. 2020 blue primary (0.131, 0.046), highlighting the color purity achievable with boron‐free architectures.

Library‐wide predictions revealed a pronounced negative correlation between *λ*_em_ and Δ*E*_ST_ (*R* = −0.75), suggesting substantial configuration mixing in the S_1_ state and providing a clear design guideline for blue TADF emitters. End‐to‐end validation—from ML‐based screening across an expansive chemical space to the experimental demonstration of narrowband blue PL and EL—supports the high reliability of our GNN surrogate model. Although predictive performance could be further improved in sparsely sampled regions through targeted expansion of the training set and active‐learning strategies, the proposed workflow is readily extensible to broader families of heteroatom‐containing PAHs and to automated synthesis pipelines.

In summary, this work expands the accessible design space of boron‐free narrowband blue emitters and provides a validated, generalizable workflow for data‐driven materials discovery. The accompanying database—nearly 20 000 enumerated structures with high‐fidelity quantum‐chemical labels for a curated subset—should serve as a valuable community resource. More broadly, this integrated strategy is expected to transfer to other data‐scarce challenges in functional organic materials, helping to shorten the design‐to‐validation cycle for next‐generation optoelectronic technologies.

## Author Contributions

**Masaya Hagai**: investigation, formal analysis, data curation, visualization, software, methodology, writing – review and editing. **Jie Sun**: investigation, formal analysis. **Jun Hyeon Lee**: investigation, formal analysis. **Kazuhiro J. Fujimoto**: conceptualization, writing – original draft, writing – review and editing, data curation, validation, supervision, funding acquisition, project administration, visualization, methodology, resources. **Shigehiro Yamaguchi**: writing – review and editing, supervision, funding acquisition. **Takuma Yasuda**: conceptualization, writing – original draft, writing – review and editing, supervision, funding acquisition, visualization, data curation, validation, resources, methodology. **Takeshi Yanai**: conceptualization, writing – review and editing, supervision, funding acquisition, resources.

## Conflicts of Interest

The authors declare no conflicts of interest.

## Supporting Information

The data that support the findings of this study are available in the Supporting Information of this article.

## Supporting information




**Supporting File**: anie73690‐sup‐0001‐SuppMat.pdf.

## Data Availability

The data that support the findings of this study are available from the corresponding author upon reasonable request.
