# Supplementary material for: Machine‐Learning‐Guided Discovery of Boron‐Free Narrowband Blue Thermally Activated Delayed Fluorescence Emitters
Source: Angew Chem Int Ed Engl. 2026 Jul 21;65(39):e2731055. doi: 10.1002/anie.2731055 (PMC13592287; doi:10.1002/anie.2731055)
Supplement: Supplementary file 1 [file ANIE-65-e2731055-s001.pdf]

Supporting Information

Machine-Learning-Guided Discovery of Boron-Free Narrowband Blue Thermally Activated Delayed Fluorescence Emitters

M. Hagai, J. Sun, J. H. Lee, K. J. Fujimoto, S. Yamaguchi, T. Yasuda*, T. Yanai**

Table of Contents

1. Enumeration and quantum-chemical characterization of 13-ring-fused carbazole-containing PAHs S3
2. Data splitting, training, and ensemble prediction S5
3. Top 50 candidates selected from GNN-guided screening S6
4. Analysis of excited state properties S11
5. Synthesis and characterization of emitters S16
6. Photophysical measurements S21
7. OLED fabrication and evaluation S25
8. Atomic coordinates of optimized Cz-PAH-1 and Cz-PAH-2 .... S26
9. References S31

1. Enumeration and quantum-chemical characterization of 13-ring-fused carbazole-containing PAHs

In this study, 13-ring-fused polycyclic aromatic hydrocarbons (PAHs) containing carbazole (Cz) motifs were exhaustively enumerated using an in-house molecular generation program implemented in Python 3.7.9 and based on RDKit 2022.03.5.[1] Molecules were represented as graphs, in which atoms and bonds correspond to nodes and edges, respectively. Ring fusion was defined as joining two cyclic subgraphs through a shared common edge.

Generation of fused-ring frameworks

Enumeration was initiated from a monocyclic scaffold consisting of either a five-membered or a six-membered ring. The scaffold was then recursively expanded by repeatedly applying a ring-fusion operation, in which an additional five- or six-membered ring was fused by sharing a selected peripheral edge. Here, a peripheral edge was defined as a bond not shared by two rings. This fusion operation was iterated until 13-ring-fused PAH skeletons composed exclusively of five- and/or six-membered rings were obtained.

To mitigate combinatorial growth and prioritize topologies expected to be more synthetically accessible, symmetry-based screening was performed at each stage where a scaffold with an odd number of fused rings was generated. Structural symmetry was evaluated using Chem.rdmolfiles.CanonicalRankAtoms; when the canonical ranks assigned by this function contained duplicate values (i.e., indicating symmetry-equivalent atoms), the scaffold was judged to possess structural symmetry. At each odd-ring stage, only symmetric skeletons were retained for subsequent growth.

All generated candidates were converted to canonical SMILES, and duplicates were removed by treating structures that yielded identical canonical SMILES as the same molecule.

Carbazole feasibility check and nitrogen substitution

Next, for each 13-ring-fused scaffold, carbazole-feasibility was evaluated and nitrogen atoms were introduced accordingly. To ensure Cz-eligible topology, every five-membered ring was required to be annulated on both sides by six-membered rings. Operationally, for each five-membered ring, at least four of the five nodes were required to belong to a six-membered ring. Whether a given node belonged to a six-membered ring was determined by checking if the corresponding atom was a member of any six-membered ring in the scaffold. When this condition was satisfied, the remaining node was substituted with a nitrogen atom to generate a carbazole substructure.

For scaffolds containing multiple five-membered rings, the same nitrogen-substitution procedure was applied to each five-membered ring such that all five-membered rings were converted into carbazole substructures. For scaffolds admitting multiple nitrogen-substitution patterns, all combinatorial substitution patterns were generated.

Chemical validity filtering (RDKit sanitization)

For each nitrogen-substituted structure, an RDKit Mol object was constructed and subjected to RDKit sanitization. Only molecules for which sanitization completed successfully—covering valence checks, aromaticity assignment, and internal consistency checks including Kekulé conversion—were retained. This filtering step ensured that only chemically valid structures were included in the final set.

In total, 19,518 unique 13-ring-fused Cz-PAH structures were generated, encoded as SMILES strings, and archived for subsequent quantum-chemical calculations and machine-learning analysis.

Quantum-chemical calculations for selected enumerated structures

From the final library of 19,518 unique 13-ring-fuzed Cz-PAHs, 1,000 molecules were randomly selected for high-accuracy quantum-chemical calculations. Initial molecular geometries were first optimized at the molecular-mechanics (MM) level and then re-optimized at the GFN-xTB[2] level. Ground-state (S_0) geometries were optimized by density functional theory (DFT) using the PBE0 functional[3] and the 6-31G(d) basis set. The resulting structures were further optimized in the first singlet excited state (S_1) using time-dependent DFT (TDDFT)[4] at the PBE0/6-31G(d) level.

The target photophysical quantities were evaluated at the appropriate optimized geometries: emission energies (λ_{em}) were calculated at the TDDFT-optimized S_1 geometries using TDDFT/PBE0/6-31G(d), whereas singlet–triplet energy gaps (ΔE_{ST}) were calculated at the DFT-optimized S_0 geometries using spin-component-scaled coupled-cluster singles and doubles at the second-order approximation (SCS-CC2)[5]/cc-pVDZ. Geometry optimizations and TDDFT calculations for emission energies were performed using the Gaussian 16 software package,[6] and SCS-CC2 calculations were performed using the TURBOMOLE version 7.6.[7] Of the 1,000 selections, 944 calculations converged successfully and were used for subsequent machine-learning model training and assessment.

Emission spectra were computed using an in-house implementation[8-9] of the thermal vibration correlation function (TVCF)[10] method, with electronic-structure inputs obtained from the TDDFT calculations.

2. Data splitting, training, and ensemble prediction

For the quantum-chemically computed subset (n = 944), the dataset was first randomly partitioned into a test set (10%) and a training/validation pool (90%). The test set was not used for training or model selection and was reserved exclusively for final evaluation. The training/validation pool was then split into training and validation subsets at a 9:1 ratio, repeated five times using different random seeds. For each split, a GNN was trained using the same model configuration and training protocol (learning rate 0.001, batch size 64, up to 300 epochs) with mean squared error (MSE) as the loss function. During training, the validation MSE was evaluated at each epoch, and the best checkpoint (the epoch with the lowest validation MSE) was selected as the final model for that split.

The GNN was based on the M3GNet architecture[11] with a Set2Set readout[12]. Since the target properties are intensive quantities, the M3GNet is_intensive option was enabled during training and inference.

The two target properties, λ_{em} and ΔE_{ST}, were learned using separate models. Specifically, two independent models with identical architectures and training settings were trained: one to predict λ_{em} and the other to predict ΔE_{ST}. For each target property, five validation-selected models were obtained, corresponding to the five training/validation splits.

For final inference, an ensemble was used for each target by taking the arithmetic mean of the predictions from the five validation-selected models. For molecule i and target $y \in \{\lambda_{\mathrm{em}}, \Delta E_{\mathrm{ST}}\}$, the ensemble prediction was computed as

$$\hat{y}_i^{(\mathrm{ens})} = \frac{1}{5}\sum_{k=1}^{5} \hat{y}_{i,k}^{(\mathrm{best})}.$$

The trained ensembles were then used to predict properties for the remaining 18,518 molecules in the 13-ring-fused Cz-PAH library.

3. Top 50 candidates selected from GNN-guided screening

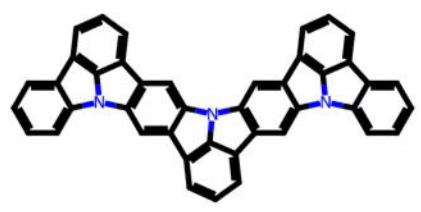

01

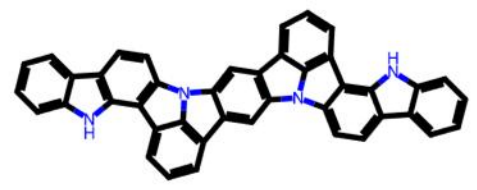

02

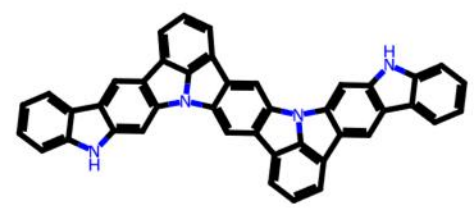

03

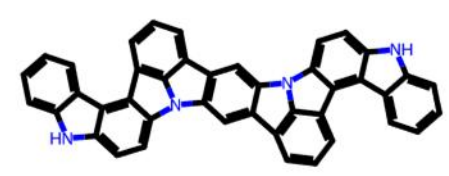

04

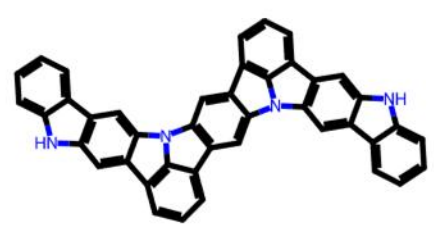

05

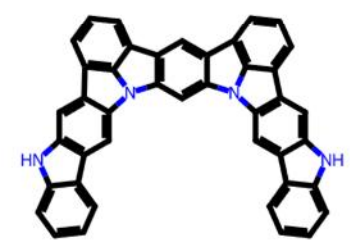

06

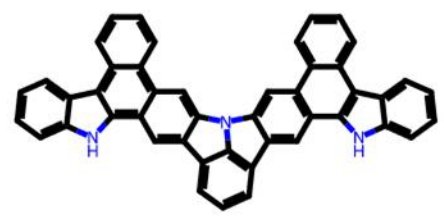

07

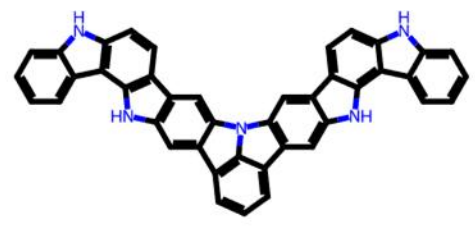

08

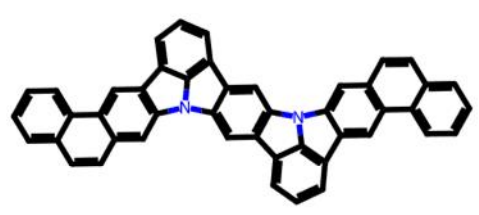

09

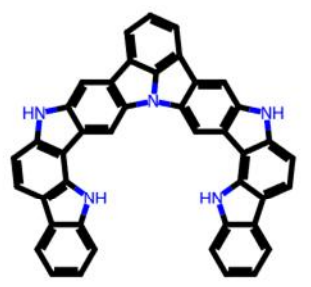

10

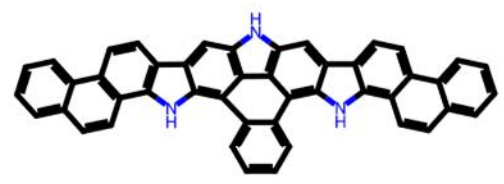

11

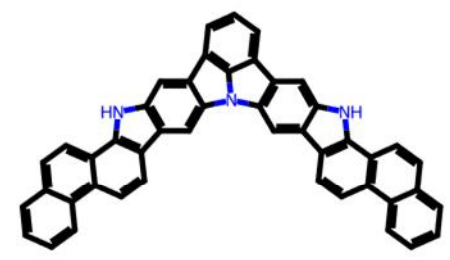

12

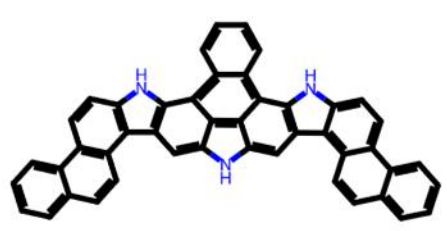

13

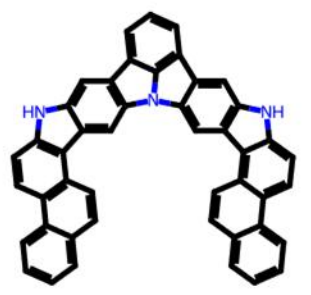

14

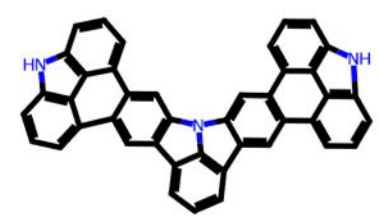

15

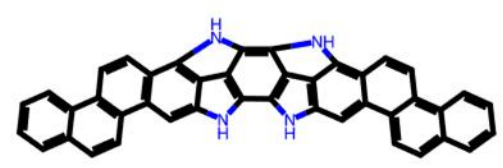

16

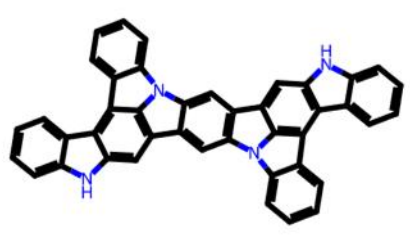

17

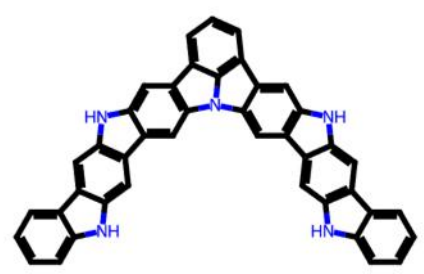

18

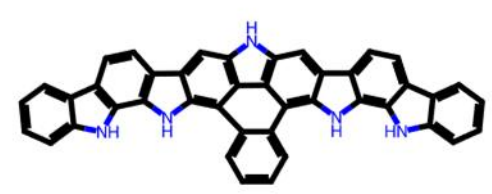

19

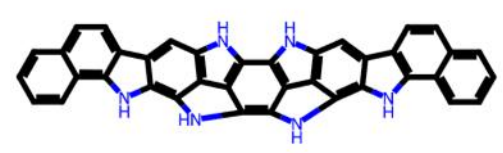

20

Figure S1.1. Candidate structures ranked 1–20 from the GNN-guided screening targeting the pure-blue region (2.4-3.0 eV), shown in order of increasing SCS-CC2[5] ΔE_{ST}.

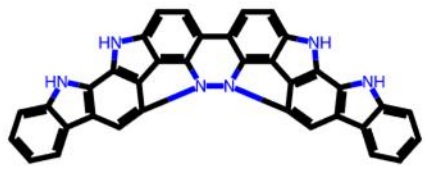

21

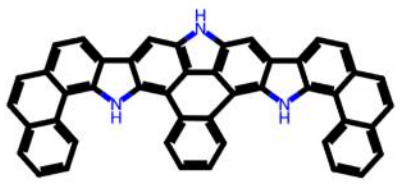

22

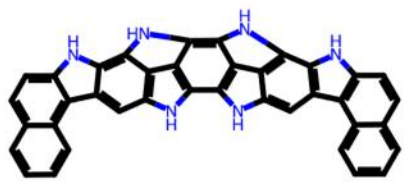

23

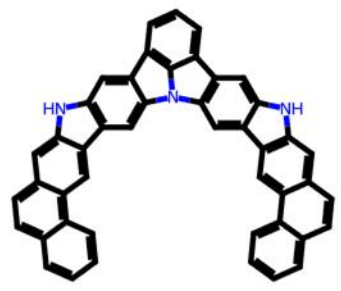

24

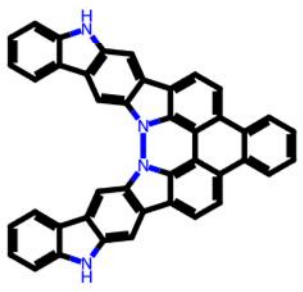

25

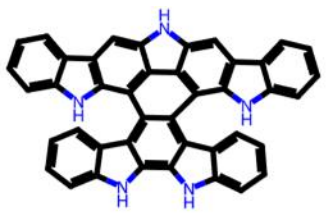

26

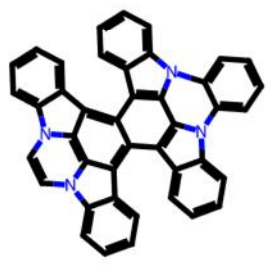

27

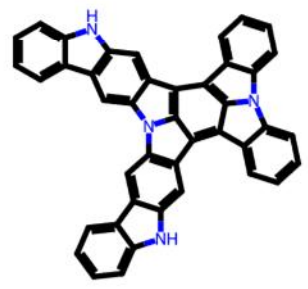

28

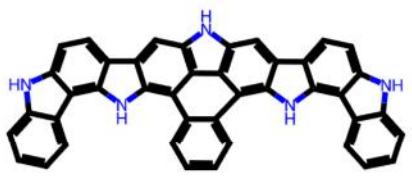

29

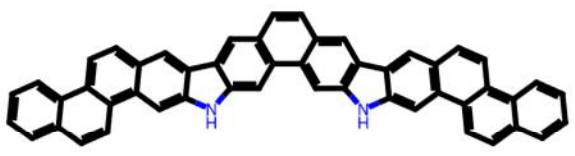

30

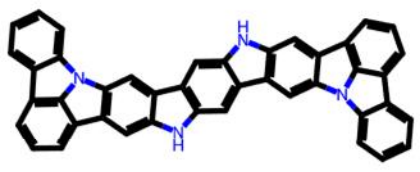

31

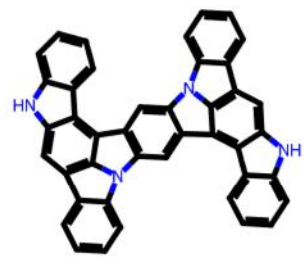

32

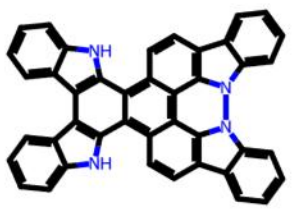

33

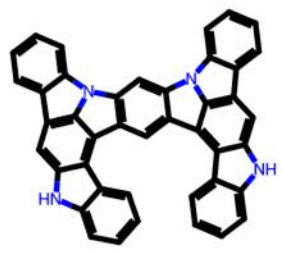

34

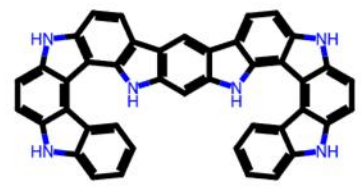

35

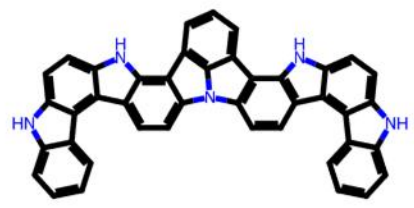

36

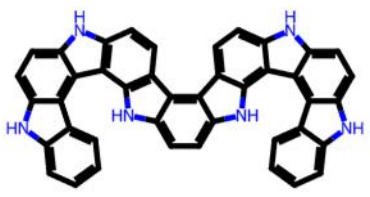

37

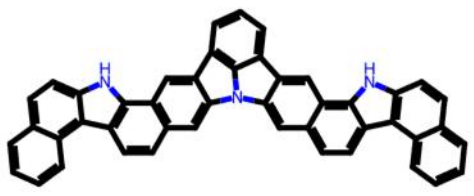

38

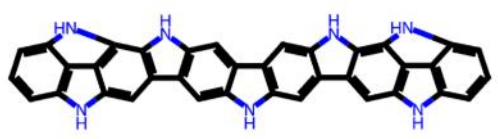

39

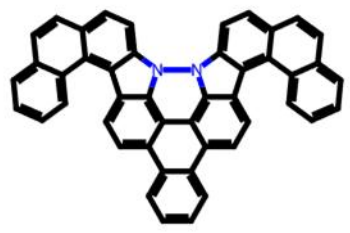

40

Figure S1.2. Candidate structures ranked 21–40 from the GNN-guided screening targeting the pure-blue region (2.4-3.0 eV), shown in order of increasing SCS-CC2[5] ΔE_{ST}.

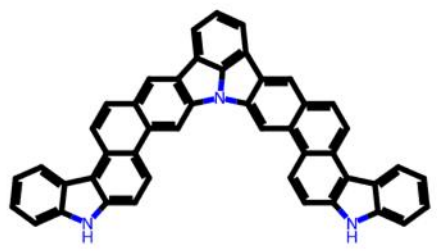

41

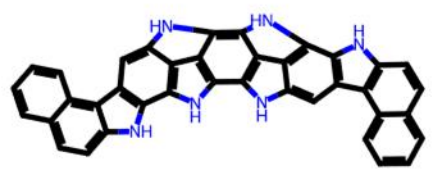

42

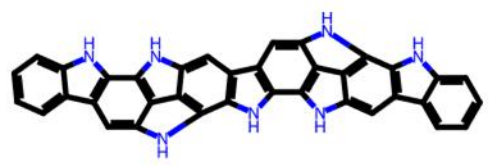

43

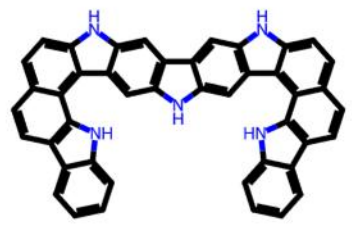

44

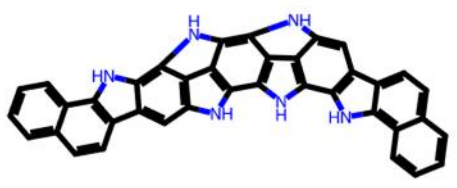

45

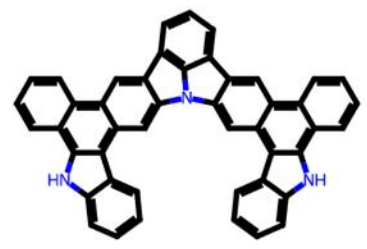

46

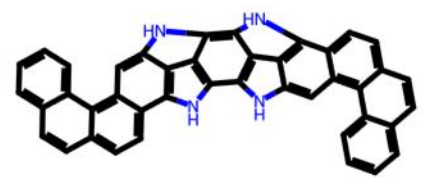

47

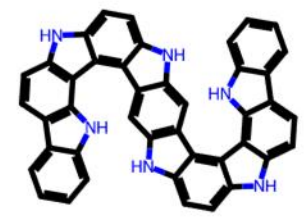

48

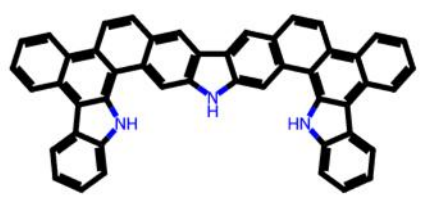

49

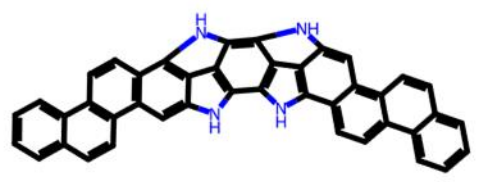

50

Figure S1.3. Candidate structures ranked 41–50 from the GNN-guided screening targeting the pure-blue region (2.4-3.0 eV), shown in order of increasing SCS-CC2[5] ΔE_{ST}.

Table S1. Calculated and predicted properties for the top 50 candidates from the GNN-guided screening. Columns 1-3 list quantum-chemical values (SCS-CC2[5] ΔE_{ST}, TDDFT[3-4] λ_{em}, and TDDFT reorganization energy Λ). Columns 4-5 list the corresponding GNN-predicted values (ΔE_{ST} and λ_{em}).

Name	ΔE_{ST} (QM) (eV)	λ_{em} (QM) (eV)	Λ (QM) (eV)	ΔE_{ST} (GNN) (eV)	λ_{em} (GNN) (eV)
01	0.13	2.76	0.12	0.30	2.84
02	0.13	2.77	0.13	0.33	2.93
03	0.16	2.90	0.12	0.33	2.94
04	0.16	2.66	0.13	0.33	2.79
05	0.16	2.69	0.11	0.28	2.80
06	0.18	3.15	0.05	0.29	2.91
07	0.19	2.73	0.10	0.29	2.77
08	0.20	2.87	0.15	0.29	3.00
09	0.20	2.86	0.09	0.32	2.79
10	0.22	2.82	0.13	0.30	2.92
11	0.23	2.83	0.08	0.33	2.91
12	0.23	2.82	0.11	0.32	2.91
13	0.23	2.76	0.08	0.31	2.79
14	0.24	2.78	0.11	0.31	2.88
15	0.24	3.00	0.11	0.30	2.89
16	0.25	2.68	0.13	0.29	2.75
17	0.25	2.70	0.07	0.27	2.88
18	0.26	2.63	0.10	0.29	2.79
19	0.26	2.84	0.13	0.32	2.90
20	0.26	2.75	0.16	0.29	2.81
21	0.26	2.54	0.27	0.33	2.51
22	0.26	2.79	0.10	0.33	2.82
23	0.26	2.61	0.15	0.30	2.76
24	0.26	2.64	0.12	0.33	2.82
25	0.27	2.46	0.14	0.31	2.52
26	0.27	2.52	0.10	0.32	2.63
27	0.28	2.54	0.11	0.30	2.71

28	0.28	2.34	0.14	0.33	2.66
29	0.29	2.95	0.09	0.32	2.90
30	0.31	2.71	0.09	0.33	2.72
31	0.31	2.74	0.07	0.32	2.84
32	0.31	2.72	0.05	0.26	2.87
33	0.32	2.73	0.09	0.31	2.45
34	0.33	2.97	0.05	0.30	2.79
35	0.33	2.83	0.24	0.30	2.97
36	0.35	2.78	0.19	0.33	2.96
37	0.35	2.90	0.22	0.32	2.88
38	0.35	2.76	0.09	0.33	2.80
39	0.36	2.79	0.10	0.30	2.81
40	0.36	2.69	0.14	0.33	2.51
41	0.37	3.03	0.09	0.31	2.89
42	0.38	2.57	0.16	0.29	2.76
43	0.39	2.86	0.15	0.27	2.83
44	-	-	-	0.31	2.84
45	0.39	2.72	0.16	0.29	2.80
46	0.40	3.15	0.09	0.28	2.75
47	0.40	2.49	0.13	0.24	2.56
48	0.42	2.73	0.10	0.30	2.99
49	0.42	2.68	0.10	0.33	2.80
50	0.44	2.59	0.13	0.28	2.76

4. Analysis of excited state properties

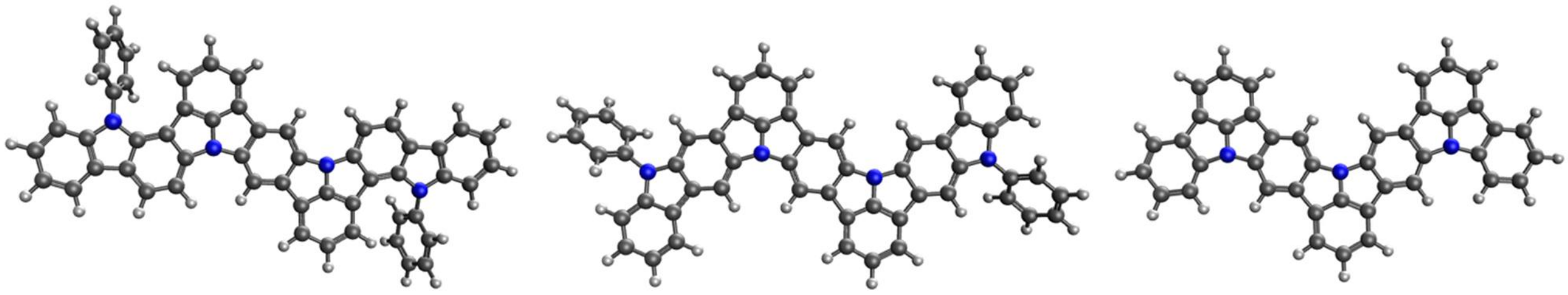

Cz-PAH-1 Cz-PAH-2 3IDCz

Figure S2. Molecular structures of Cz-PAH-1, Cz-PAH-2, and the structurally related 3IDCz reference molecule. Cz-PAH-1 and Cz-PAH-2 were synthesized in this study, whereas 3IDCz was previously reported by Lee et al.[13]

Table S2. Calculated excited-state properties of Cz-PAH-1, Cz-PAH-2, and 3IDCz. Vertical excitation energies of the S_1 and T_1 states, the singlet–triplet energy gap ΔE_{ST}, and the oscillator strength $f(S_1)$ are summarized. All calculations were performed using geometries optimized at the DFT PBE0/6-31G(d) level.

Molecule	Method	$E(S_1)$ (eV)	$E(T_1)$ (eV)	ΔE_{ST} (eV)	$f(S_1)$
Cz-PAH-1	TD-PBE0/ 6-31G(d)	3.07	2.61	0.46	0.37
	SCS-CC2/ cc-pVDZ	3.28	3.16	0.13	0.48
Cz-PAH-2	TD-PBE0/ 6-31G(d)	2.88	2.50	0.38	0.14
	SCS-CC2/ cc-pVDZ	3.11	2.95	0.16	0.19
3IDCz	TD-PBE0/ 6-31G(d)	2.98	2.59	0.40	0.16
	SCS-CC2/ cc-pVDZ	3.20	3.07	0.13	0.23

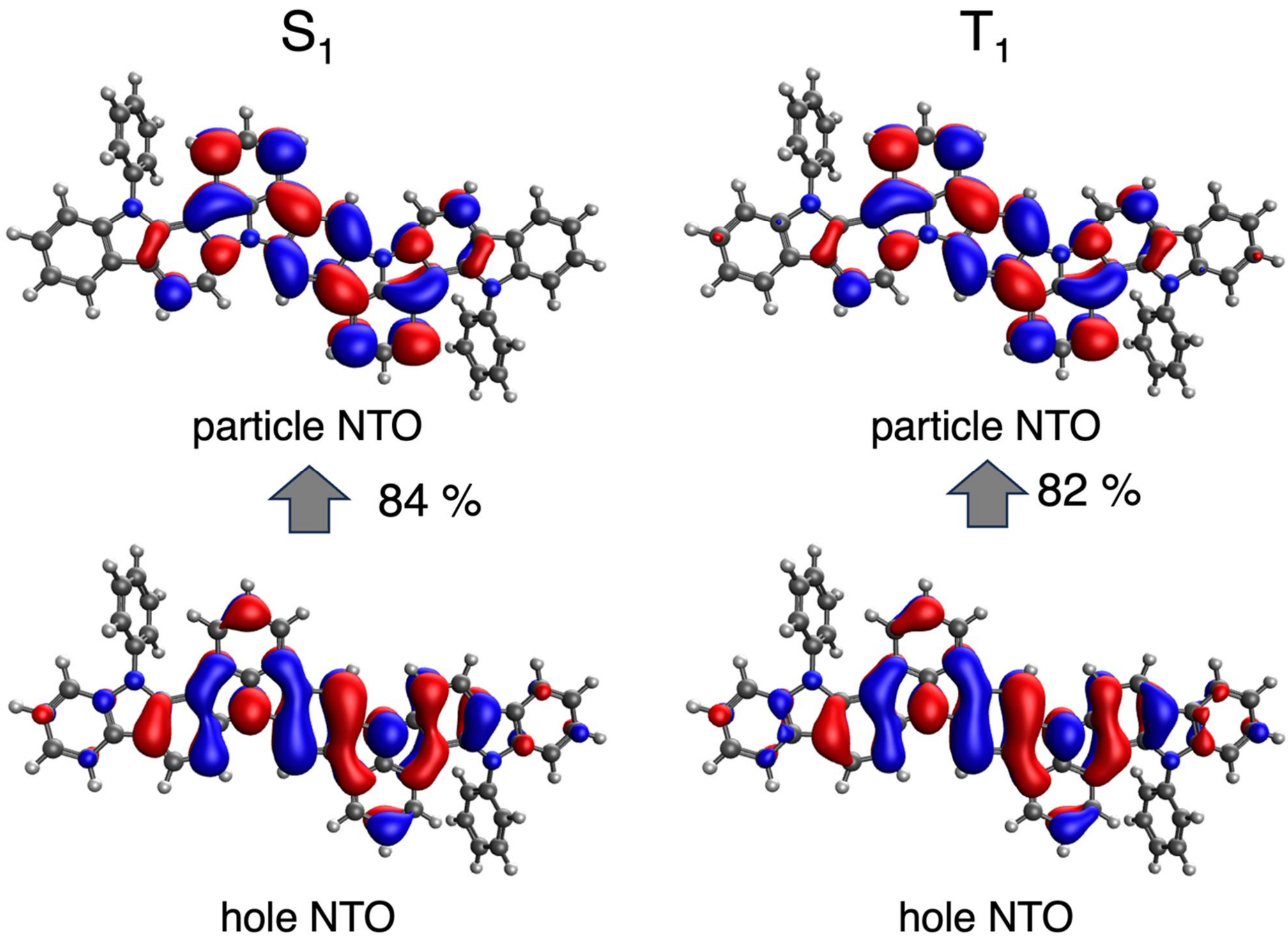

Figure S3. Natural transition orbitals (NTOs) of the S_1 and T_1 states of Cz-PAH-1 calculated at the SCS-CC2/cc-pVDZ level. The isosurface value is 0.05 a.u. The left and right panels show the dominant hole–particle NTO pairs of the S_1 and T_1 states, accounting for 84% and 82% of the corresponding excitations, respectively. The hole and particle NTOs exhibit partially complementary distributions over the π-conjugated core, consistent with multi-resonance-type excitation character.

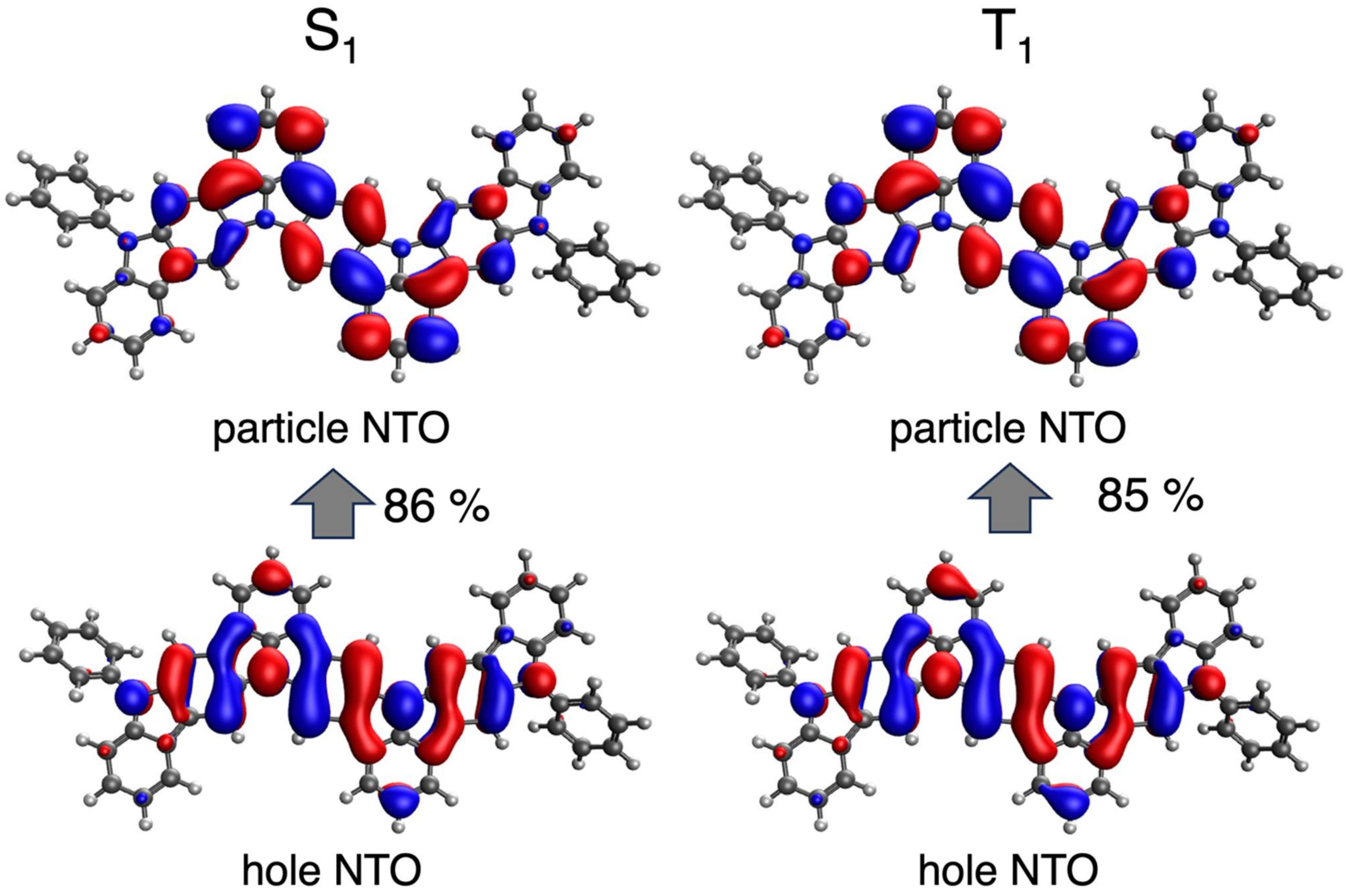

Figure S4. NTOs of the S_1 and T_1 states of Cz-PAH-2 calculated at the SCS-CC2/cc-pVDZ level. The isosurface value is 0.05 a.u. The left and right panels show the dominant hole–particle NTO pairs of the S_1 and T_1 states, accounting for 86% and 85% of the corresponding excitations, respectively. The hole and particle NTOs exhibit partially complementary distributions over the π-conjugated core, consistent with multi-resonance-type excitation character.

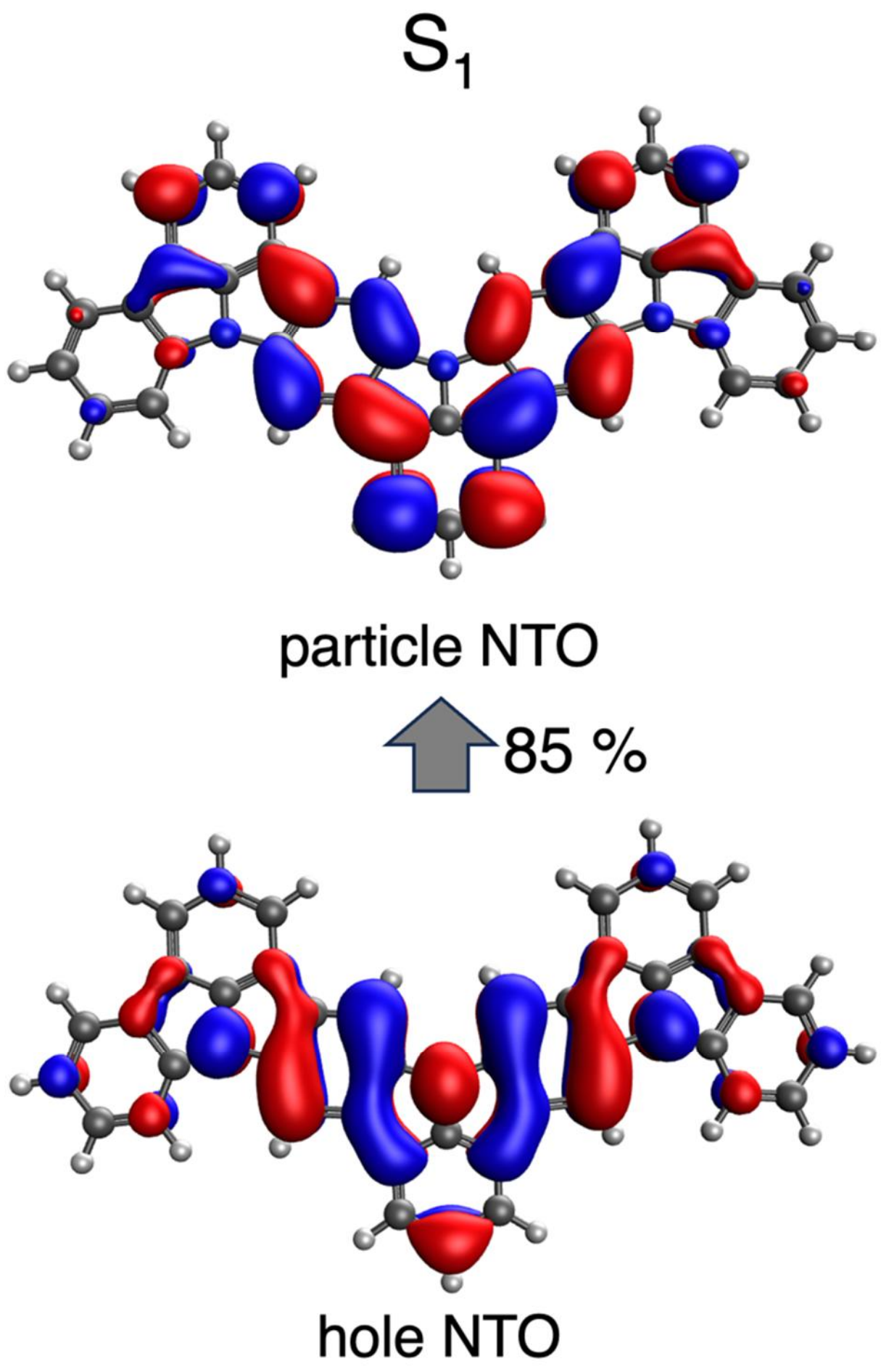

Figure S5. NTOs of the S_1 and T_1 states of 3IDCz calculated at the SCS-CC2/cc-pVDZ level. The isosurface value is 0.05 a.u. The left and right panels show the dominant hole–particle NTO pairs of the S_1 and T_1 states, accounting for 85% and 87% of the corresponding excitations, respectively. The hole and particle NTOs exhibit partially complementary distributions over the π-conjugated core, consistent with multi-resonance-type excitation character.

Table S3. Excitation characters of the S_1 and T_1 states of Cz-PAH-1 and Cz-PAH-2 calculated at the SCS-CC2/cc-pVDZ level. Dominant one-electron transition configurations with contributions larger than 1% are listed. The single- and double-excitation contributions correspond to the %t1 and %t2 values reported by TURBOMOLE, respectively. All calculations were performed using geometries optimized at the DFT PBE0/6-31G(d) level.

Molecule	State	Main transition	Other contributions > 1%	Single	Double
Cz-PAH-1	S_1	HOMO→LUMO (77.5%)	HOMO − 4 → LUMO+1 (5.9%)<br>HOMO − 5 → LUMO (4.0%)<br>HOMO − 7 → LUMO (2.0%)<br>HOMO − 3 → LUMO+9 (1.3%)	87.36%	12.64%
	T_1	HOMO→LUMO (76.5%)	HOMO − 4 → LUMO+1 (6.0%)<br>HOMO − 5 → LUMO (3.6%)<br>HOMO − 7 → LUMO (3.2%)<br>HOMO − 2 → LUMO+1 (1.7%)<br>HOMO − 5 → LUMO+2 (1.3%)	88.72%	11.28%
Cz-PAH-2	S_1	HOMO→LUMO (79.3%)	HOMO−3 → LUMO+1 (7.4%)<br>HOMO−5 → LUMO (2.1%)<br>HOMO−5 → LUMO+2 (1.2%)	87.15%	12.85%
	T_1	HOMO→LUMO (77.5%)	HOMO−3 → LUMO+1 (10.7%)<br>HOMO−5 → LUMO (2.2%)<br>HOMO−5 → LUMO+2 (2.1%)	88.39%	11.61%

5. Synthesis and characterization of emitters

The synthetic routes for **Cz-PAH-1** and **Cz-PAH-2** are outlined in Schemes S1 and S2, respectively. Detailed synthetic procedures and characterization data are described below. All chemical reactions were performed under a dry N_2 atmosphere using anhydrous solvents unless otherwise noted.

Nuclear magnetic resonance (NMR) spectra were recorded on an Avance III 400 spectrometer (Bruker). ^{1}H NMR chemical shifts (δ) were determined relative to tetramethylsilane (δ = 0.00) and the residual solvent signals of $CDCl_3$ (δ = 77.0) and DMSO-d_6 (δ = 2.50). ^{13}C{^{1}H} NMR spectra are referenced to the solvent signals of $CDCl_3$ (δ = 77.16). Matrix-assisted laser desorption ionization time-of-flight (MALDI-TOF) mass spectra were collected on an Autoflex III spectrometer (Bruker Daltonics) using dithranol as a matrix. High resolution mass spectrometric (HRMS) data were obtained using a micrOTOF-Q III (Bruker) at an ambient temperature. Thermogravimetric analysis (TGA) was performed on a TG/DTA7300 analyzer (Hitachi High-Tech Science) under N_2.

Synthesis of Cz-PAH-1:

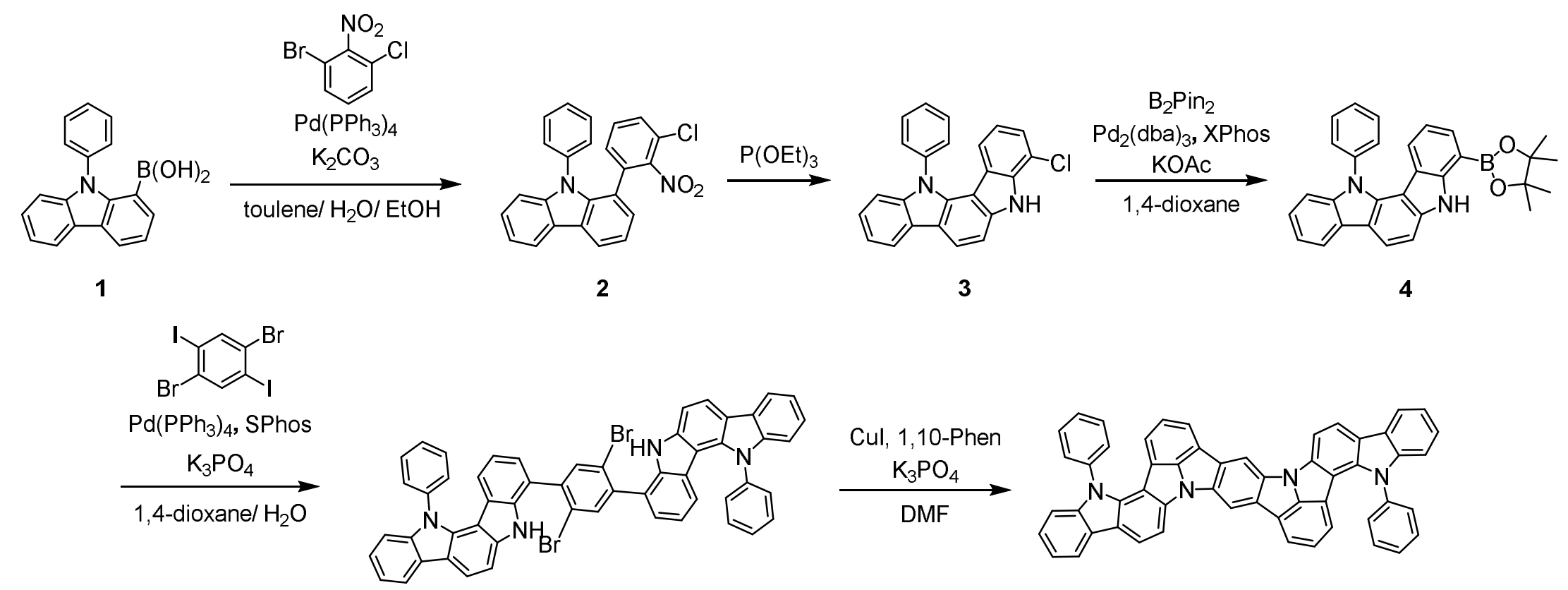

Scheme S1. Synthesis of **Cz-PAH-1**.

1-(3-Chloro-2-nitrophenyl)-9-phenyl-9*H*-carbazole (2): A mixture of **1** (2.87 g, 10.0 mmol), 1-bromo-3-chloro-2-nitrobenzene (4.73 g, 20.0 mmol), K_2CO_3 (2.76 g, 20.0 mmol), and $Pd(PPh_3)_4$ (0.58 g, 0.5 mmol) in toluene, EtOH, and water (2:1:1, v/v/v) was purged with N_2 for 10 min. The mixture was refluxed for 16 h. After cooling to room temperature, the reaction mixture was added into water and then extracted with dichloromethane. The combined organic layer was washed with water and dried over anhydrous Na_2SO_4. After filtration and evaporation, the crude product was purified by silica gel column chromatography (eluent: hexane/CH_2Cl_2 = 4:1, v/v) to afford **2** (2.23 g, 56%) as a yellow solid. ^{1}H NMR (400 MHz, $CDCl_3$): δ 8.26 (dd, J = 7.7, 1.4 Hz, 1H), 8.20 (dd, J = 7.6, 1.2 Hz, 1H), 7.42 (t, J = 8.4 Hz, 1H), 7.39–7.30 (m, 4H), 7.28–7.16 (m, 5H), 7.09 (t, J = 7.7 Hz, 1H), 6.85 (t, J = 7.9 Hz, 1H), 6.79 (dd, J = 7.8, 1.4 Hz, 1H). ^{13}C{^{1}H} NMR (100 MHz, $CDCl_3$): δ 130.56, 129.91, 129.35, 129.02, 128.64, 128.55, 127.65, 127.54, 126.45, 124.95, 124.37, 123.47,

122.70, 121.92, 121.34, 120.42, 120.13, 119.58, 119.53, 119.35, 110.15, 104.56. MS (MALDI-TOF): *m*/*z* calcd 398.08 $[M]^+$; found 398.02.

4-Chloro-12-phenyl-5,12-dihydroindolo[3,2-a]carbazole (3): A solution of **2** (2.18 g, 5.46 mmol) in triethyl phosphite (20 mL) was stirred at 180 °C for 24 h. After cooling to room temperature, the reaction mixture was added into water and then extracted with chloroform. The combined organic layer was washed with water and dried over anhydrous Na_2SO_4. After filtration and evaporation, the crude product was purified by silica gel column chromatography (eluent: hexane/CH_2Cl_2 = 3:1, v/v) to afford **3** (0.40 g, 20%) as a white solid. ^{1}H NMR (400 MHz, $CDCl_3$): δ 8.62 (s, 1H), 8.28 (dd, J = 8.4, 1.4 Hz, 1H), 8.24–8.14 (d, J = 7.6 Hz, 1H), 7.70–7.60 (m, 5H), 7.54–7.47 (d, J = 7.6 Hz, 1H), 7.45–7.31 (m, 3H), 7.34–7.25 (m, 1H), 6.71 (t, J = 7.9 Hz, 1H), 5.77 (d, J = 8.2 Hz, 1H). ^{13}C{^{1}H} NMR (100 MHz, $CDCl_3$): δ 141.71, 140.17, 139.80, 136.82, 135.89, 129.91, 129.35, 128.54, 124.37, 124.30, 123.47, 123.09, 121.92, 120.48, 119.57, 119.34, 118.92, 117.36, 115.40, 110.16, 108.28, 104.56. MS (MALDI-TOF): *m*/*z* calcd 366.09 $[M]^+$; found 365.98.

12-Phenyl-4-(4,4,5,5-tetramethyl-1,3,2-dioxaborolan-2-yl)-5,12-dihydroindolo[3,2-a]carbazole (4): A mixture of **3** (0.83 g, 2.25 mmol), bis(pinacolato)diboron (B_2Pin_2, 1.14 g, 4.5 mmol), $Pd_2(dba)_3$ (0.10 g, 0.11 mmol), XPhos (0.11 g, 0.23 mmol), KOAc (0.88 g, 9.0 mmol) in dry 1,4-dioxane (5 mL) was stirred for 16 h at 100 °C. After cooling to room temperature, the reaction mixture was added into water and then extracted with dichloromethane. The combined organic layer was washed with water and dried over anhydrous Na_2SO_4. After filtration and evaporation, the crude product was purified by silica gel column chromatography (eluent: hexane/ CH_2Cl_2 = 2:1, v/v) to afford **4** (0.60 g, 58%) as a yellowish white solid. ^{1}H NMR (400 MHz, $CDCl_3$): δ 9.58 (s, 1H), 8.26 (d, J = 8.4 Hz, 1H), 8.21–8.14 (m, 1H), 7.73 (dd, J = 7.1, 1.2 Hz, 1H), 7.65 (s, 5H), 7.54 (d, J = 8.4 Hz, 1H), 7.43–7.31 (m, 3H), 6.79 (t, J = 8.1, 1H), 6.00 (d, J = 8.0 Hz, 1H), 1.46 (s, 12H). ^{13}C{^{1}H} NMR (100 MHz, $CDCl_3$): δ 144.18, 141.64, 140.37, 139.90, 137.00, 131.39, 129.82, 129.28, 128.31, 127.00, 124.60, 124.01, 120.59, 120.29, 118.75, 118.48, 118.41, 116.72, 110.06, 104.55, 84.01, 25.03. (Two aromatic signal could not be observed because of the overlapping). MS (MALDI-TOF): *m*/*z* calcd 458.22 $[M]^+$; found 458.18.

4,4'-(2,5-Dibromo-1,4-phenylene)bis(12-phenyl-5,12-dihydroindolo[3,2-a]carbazole) (5): A mixture of **4** (0.69 g, 1.5 mmol), 1,4-dibromo-2,5-diiodobenzene (0.37 g, 0.75 mmol), $Pd(PPh_3)_4$ (0.04 g, 0.04 mmol), SPhos (0.03 g, 0.08 mmol) in K_3PO_4 aq. (4 M, 3 mL) and 1,4-dioxane (6 mL) was stirred for 2 h at 100 °C. After cooling to room temperature, the reaction mixture was added into water and then extracted with chloroform. The combined organic layer was washed with water and dried over anhydrous Na_2SO_4. After filtration and evaporation, the crude product was purified by silica gel column chromatography (eluent: hexane/ $CHCl_3$ = 2:1, v/v) to afford **5** (0.59 g, 88%) as a white solid. ^{1}H NMR (400 MHz, DMSO-d_6): δ 11.49 (s, 1H), 8.27 (dd, J = 8.4, 7.9 Hz, 4H), 7.91 (s, 2H), 7.84–7.73 (m, 8H), 7.70 (s, 4H), 7.55 (d, J = 8.5 Hz, 2H), 7.40–7.28 (m, 4H), 7.21 (m, 4H), 6.80 (t, J = 7.7 Hz, 2H), 5.86 (d, J = 8.1 Hz, 2H). ^{13}C NMR spectrum could not be obtained because of its low solubility. MS (MALDI-TOF): *m*/*z* calcd 896.10 $[M]^+$; found 896.15.

Cz-PAH-1: A mixture of **5** (0.36 g, 0.4 mmol), CuI (0.015 g, 0.08 mmol), 1,10-phenanthroline (0.029 g, 0.16 mmol), K_3PO_4 (0.42 g, 2.0 mmol) in DMF (2 mL) was stirred for 3 h at 100 °C. After cooling to room temperature, the reaction mixture was quenched by the addition of MeOH. After filtration, the crude product was successively washed with hot toluene to afford **Cz-PAH-1** (0.175 g, 58%) as a yellowish white solid. NMR spectra could not be obtained because of its low solubility. HRMS: *m*/*z* calcd 734.247 $[M]^+$; found 734.247.

Synthesis of Cz-PAH-2:

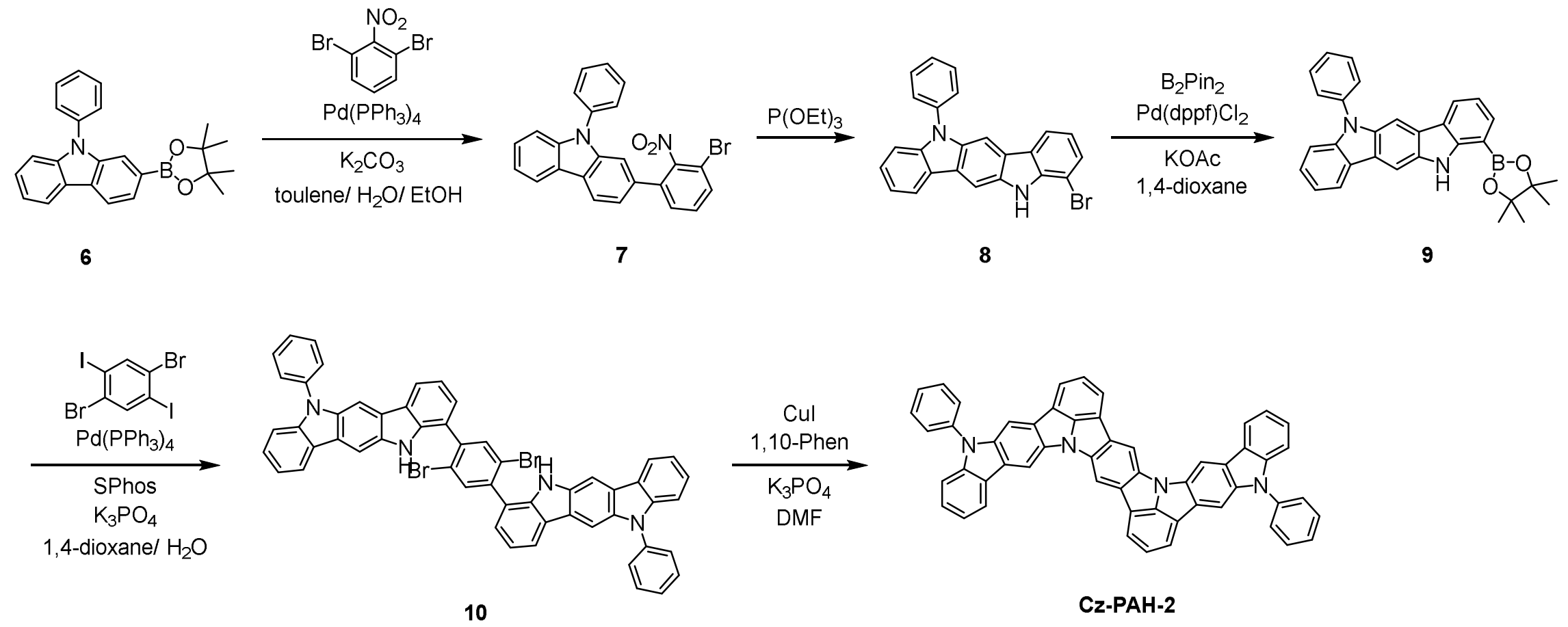

Scheme S2. Synthesis of **Cz-PAH-2**.

2-(3-Bromo-2-nitrophenyl)-9-phenyl-9*H*-carbazole (7): A mixture of **6** (3.69 g, 10.0 mmol), 1,3-dibromo-2-nitrobenzene (2.81 g, 10.0 mmol), K_2CO_3 (2.76 g, 20.0 mmol), and $Pd(PPh_3)_4$ (0.58 g, 0.5 mmol) in toluene, EtOH, and water (2:1:1, v/v/v) was purged with N_2 for 10 min. The mixture was refluxed for 16 h. After cooling to room temperature, the reaction mixture was added into water and then extracted with dichloromethane. The combined organic layer was washed with water and dried over anhydrous Na_2SO_4. After filtration and evaporation, the crude product was purified by silica gel column chromatography (eluent: hexane/CH_2Cl_2 = 4:1, v/v) to afford **7** (3.94 g, 89%) as a yellow solid. ^{1}H NMR (400 MHz, DMSO-d_6): δ 8.38 (dd, J = 8.0, 0.7 Hz, 1H), 8.33 (dt, J = 7.8, 1.1 Hz, 1H), 7.93 (dd, J = 8.0, 1.3 Hz, 1H), 7.76–7.68 (m, 3H), 7.67–7.60 (m, 3H), 7.60–7.54 (m, 1H), 7.51 (ddd, J = 8.3, 7.0, 1.2 Hz, 1H), 7.43 (dt, J = 8.3, 1.0 Hz, 1H), 7.40–7.27 (m, 3H). ^{13}C{^{1}H} NMR (100 MHz, $CDCl_3$): δ 141.52, 140.74, 137.21, 137.02, 132.99, 132.22, 130.73, 130.43, 130.01, 127.72, 127.04, 126.64, 123.90, 122.77, 120.77, 120.61, 120.37, 119.93, 113.13, 110.00, 109.63, 77.22.MS (MALDI-TOF): *m*/*z* calcd 442.02 $[M]^+$; found 442.03.

4-Bromo-11-phenyl-5,11-dihydroindolo[3,2-b]carbazole (8): A solution of **7** (2.98 g, 6.7 mmol) in triethyl phosphite (25 mL) was stirred at 180 °C for 24 h. After cooling to room temperature, the reaction mixture was added into water and then extracted with chloroform. The combined organic layer was washed with water and dried over anhydrous Na_2SO_4. After filtration and evaporation, the crude product was purified by silica gel column chromatography (eluent: hexane/CH_2Cl_2 = 3:1, v/v)

to afford **8** (0.75 g, 27%) as a white solid. ^{1}H NMR (400 MHz, DMSO-d_6): δ 11.40 (s, 1H), 8.36 (d, *J* = 7.9 Hz, 1H), 8.32 (d, *J* = 0.9 Hz, 1H), 8.23 (d, *J* = 7.7 Hz, 1H), 8.10 (s, 1H), 7.74 (d, *J* = 5.7 Hz, 3H), 7.66–7.56 (m, 3H), 7.45 (dd, *J* = 6.9, 1.3 Hz, 1H), 7.38 (d, *J* = 8.1 Hz, 1H), 7.32 – 7.28 (m, 1H), 7.06 (t, *J* = 7.7 Hz, 1H). ^{13}C{^{1}H} NMR (100 MHz, $CDCl_3$) δ 140.89, 138.72, 138.58, 130.15, 128.52, 127.90, 126.44, 125.24, 125.11, 125.06, 124.70, 123.93, 122.40, 121.61, 120.35, 120.11, 119.99, 119.81, 112.76, 112.41, 110.84, 109.68. MS (MALDI-TOF): *m*/*z* calcd 410.04 [*M*]$^+$; found 409.96.

11-Phenyl-4-(4,4,5,5-tetramethyl-1,3,2-dioxaborolan-2-yl)-5,11-dihydroindolo[3,2-b]carbazole (9): A mixture of **8** (0.74 g, 1.8 mmol), B_2Pin_2 (0.68 g, 2.7 mmol), Pd(dppf)Cl_2 (0.07 g, 0.1 mmol), KOAc (0.35 g, 3.6 mmol) in dry 1,4-dioxane (10 mL) was stirred for 15 h at 100 °C. After cooling to room temperature, the reaction mixture was added into water and then extracted with dichloromethane. The combined organic layer was washed with water and dried over anhydrous Na_2SO_4. After filtration and evaporation, the crude product was purified by silica gel column chromatography (eluent: hexane/CH_2Cl_2 = 2:1, v/v) to afford **9** (0.48 g, 58%) as a yellowish white solid. ^{1}H NMR (400 MHz, $CDCl_3$): *δ* 9.24 (s, 1H), 8.29 – 8.19 (m, 3H), 8.08 (s, 1H), 7.92 (dd, *J* = 7.2, 1.3 Hz, 1H), 7.77–7.66 (m, 4H), 7.59–7.52 (m, 1H), 7.52–7.43 (m, 2H), 7.33 (t, *J* = 8.0, 1H), 7.25 (t, *J* = 7.4 Hz, 1H), 1.51 (s, 12H). ^{13}C{^{1}H} NMR (100 MHz, $CDCl_3$): *δ* 146.59, 142.02, 138.47, 136.76, 135.39, 132.96, 129.98, 127.33, 127.21, 125.90, 123.65, 123.36, 123.17, 122.50, 120.15, 119.28, 118.13, 109.52, 100.61, 99.96, 84.02, 25.11. MS (MALDI-TOF): *m*/*z* calcd 458.22 [*M*]$^+$; found 458.53.

10,10'-(2,5-Dibromo-1,4-phenylene)bis(5-phenyl-5,11-dihydroindolo[3,2-b]carbazole) (10): A mixture of **9** (0.92 g, 2.0 mmol), 1,4-dibromo-2,5-diiodobenzene (0.49 g, 1.0 mmol), Pd(PPh_3)$_4$ (0.058 g, 0.05 mmol), SPhos (0.041 g, 0.1 mmol) in K_3PO_4 aq. (4 M, 4 mL) and 1,4-dioxane (8 mL) was stirred for 3 h at 100 °C. After cooling to room temperature, the reaction mixture was added into water and then extracted with chloroform. The combined organic layer was washed with water and dried over anhydrous Na_2SO_4. After filtration and evaporation, the crude product was purified by silica gel column chromatography (eluent: hexane/$CHCl_3$ = 2:1, v/v) to afford **10** (0.36 g, 40%) as a white solid. ^{1}H NMR (400 MHz, DMSO-d_6) δ 11.14 (s, 1H), 8.44–8.23 (m, 6H), 8.17 (s, 2H), 8.01 (s, 2H), 7.77 (d, *J* = 3.3 Hz, 8H), 7.61 (s, 2H), 7.51–7.35 (m, 6H), 7.30 (t, *J* = 7.5 Hz, 4H). ^{13}C NMR spectrum could not be obtained because of its low solubility. MS (MALDI-TOF): *m*/*z* calcd 896.10 [*M*]$^+$; found 896.39.

Cz-PAH-2: A mixture of **10** (0.32 g, 0.36 mmol), CuI (0.014 g, 0.07 mmol), 1,10-phenanthroline (0.026 g, 0.14 mmol), K_3PO_4 (0.38 g, 1.8 mmol) in DMF (2 mL) was stirred for 3 h at 100 °C. After cooling to room temperature, the reaction mixture was quenched by the addition of MeOH. After filtration, the crude product was successively washed with hot toluene to afford **Cz-PAH-2** (0.086 g, 33%) as a yellowish white solid. NMR spectra could not be obtained because of its low solubility. HRMS: *m*/*z* calcd 734.247 [*M*]$^+$; found 734.249.

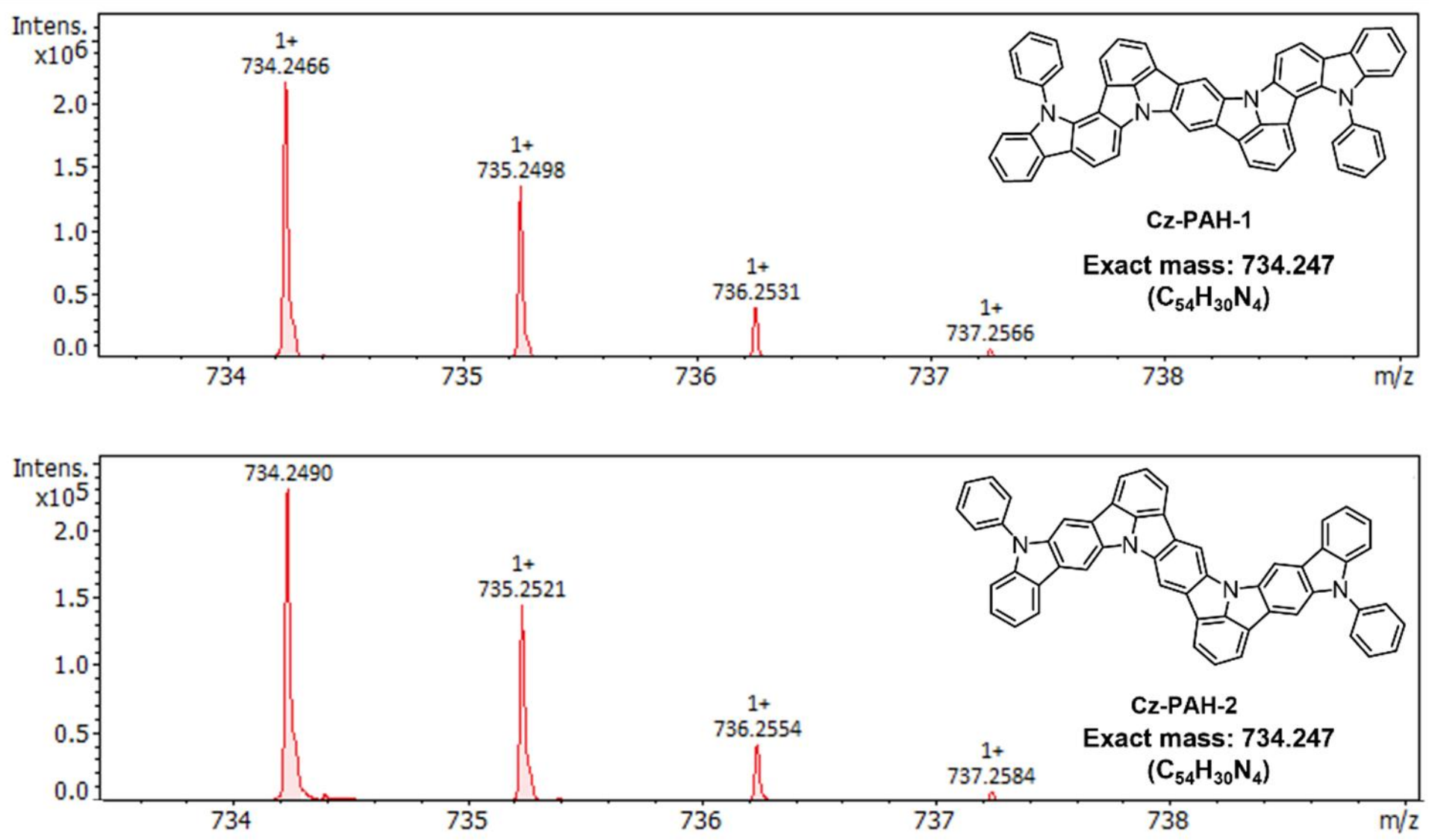

Figure S6. High resolution mass (HRMS) spectra of **Cz-PAH-1** (top) and **Cz-PAH-2** (bottom).

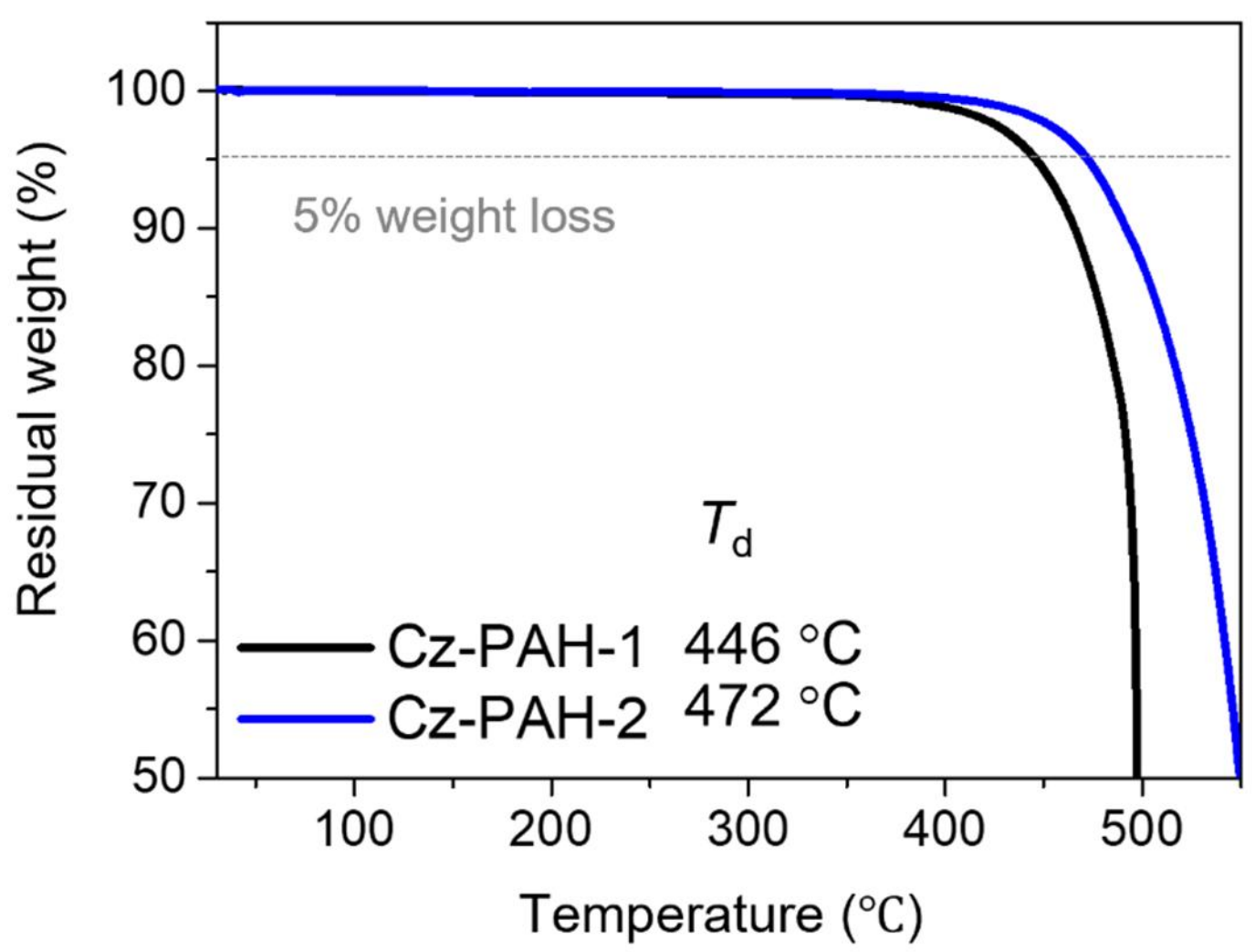

Figure S7. TGA thermograms of **Cz-PAH-1** and **Cz-PAH-2** recorded under N_2 at a heating rate of 10 °C min^{-1} (T_d: 5% weight-loss temperature).

6. Photophysical measurements

Thin-film samples for photophysical measurements were deposited onto quartz substrates by vacuum deposition (<9 × 10^{-5} Pa) using an E-200 vacuum evaporation system (ALS Technology). UV−vis absorption and PL spectra were recorded on a V-670 spectrometer (Jasco) and an FP-8600 spectrophotometer (Jasco), respectively. The absolute PL quantum yields were determined using a C13534 integrating sphere system (Hamamatsu Photonics). The transient PL decay measurements were performed on a C11367 Quantaurus-tau fluorescence lifetime spectrometer (Hamamatsu Photonics; λ_{ex} = 280 nm, pulse width = 100 ps, and repetition rate = 5 kHz) under N_2 for solution samples or under vacuum (<4 × 10^{-1} Pa) for thin-film samples. The emission lifetimes were determined from the PL decay curves by fitting with exponential functions and deconvoluting with the instrument response function. The photophysical rate constants for radiative decay (k_r), intersystem crossing (k_{ISC}), and reverse intersystem crossing (k_{RISC}) were calculated according to the literature method[14] (see Tables S4 and S5).

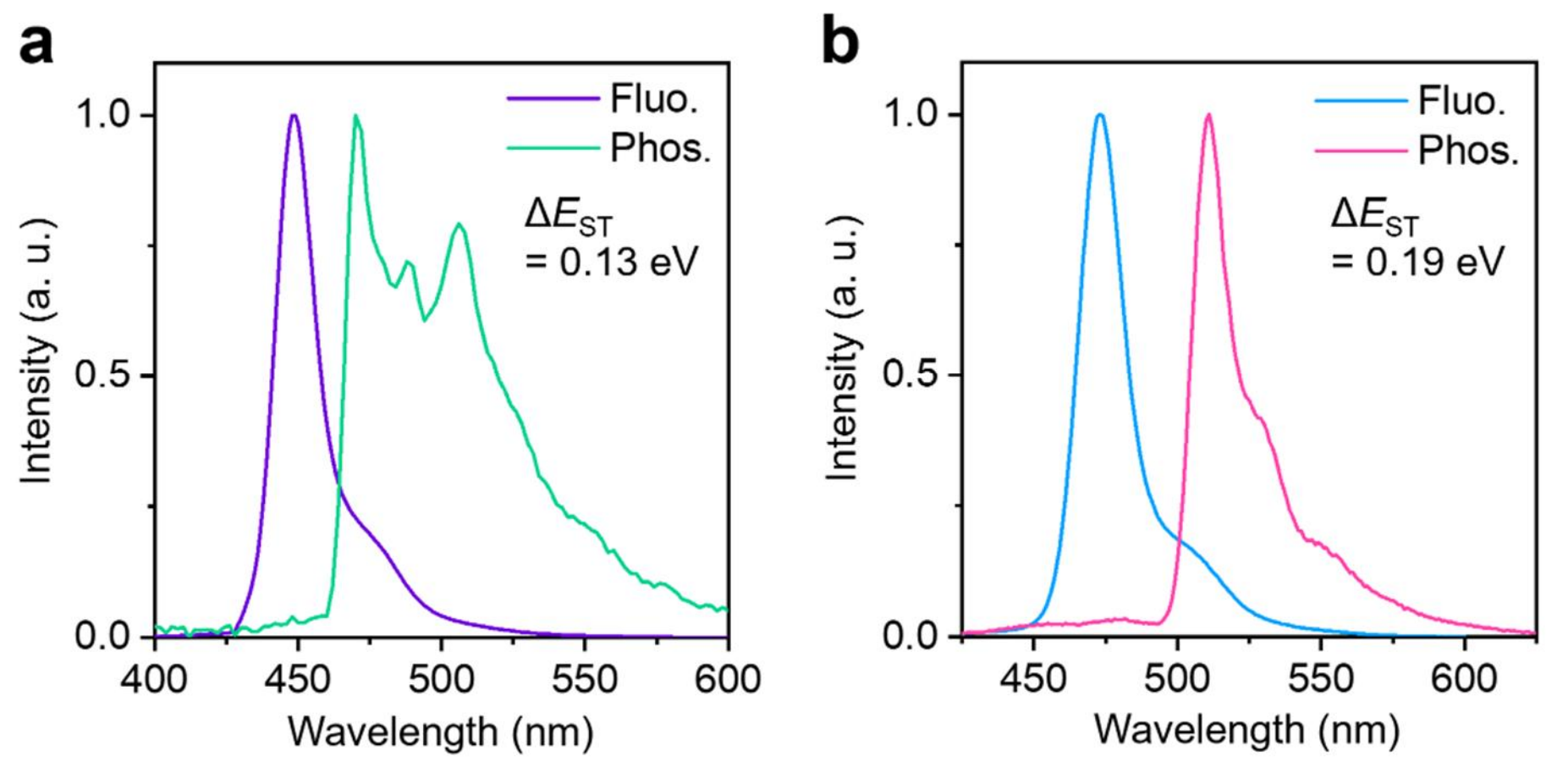

Figure S8. Fluorescence (300 K) and phosphorescence (77 K) spectra of (a) **Cz-PAH-1** and (b) **Cz-PAH-2** in toluene (10^{-5} M).

Table S4. Summary of photophysical properties.

Emitter	State[a]	λ_{em}[b] (nm)	FWHM[c] (nm/eV)	Φ_{PL}[d] (%)	Φ_p[e] (%)	Φ_d[e] (%)	τ_p[f] (ns)	τ_d[f] (μs)	ΔE_{ST}[g] (eV)
**Cz-PAH-1**	Sol	448	17/0.10	92	92	~0	6.0	–	0.13
	Film A	457	21/0.12	93	90	3	5.0	22	0.21
	Film B	457	23/0.13	99	79	20	6.5	1.9	–
**Cz-PAH-2**	Sol	473	19/0.10	86	86	~0	15	–	0.19
	Film A	482	23/0.12	99	89	10	14	15	0.16
	Film B	483	25/0.13	~100	92	8	16	3.0	–

[a]Sol = deoxygenated toluene solution (10^{-5} M); Film A = Cz-PAH:mCPBC (1:99, w/w) binary doped film; Film B = Cz-PAH:p4TCzPhBN:mCPBC (1:20:79, w/w/w) ternary doped film. [b]PL emission maximum. [c]Full width at half maximum of the PL spectrum given in wavelength and energy. [d]Absolute PL quantum yield evaluated using an integrating sphere. [e]Fractional quantum yields for prompt fluorescence (Φ_p) and delayed fluorescence (Φ_d); $\Phi_p + \Phi_d = \Phi_{PL}$. [f]Emission lifetimes for prompt fluorescence (τ_p) and delayed fluorescence (τ_d). [g]Singlet–triplet energy gap estimated from the fluorescence and phosphorescence emission peak positions.

Table S5. Summary of rate constants.

Emitter	State[a]	k_r[b] (10^7 s^{-1})	k_{ISC}[c] (10^6 s^{-1})	k_{RISC}[d] (10^4 s^{-1})
**Cz-PAH-1**	Sol	15	–	–
	Film A	18	17	1.0
	Film B	12	32	63
**Cz-PAH-2**	Sol	5.6	–	–
	Film A	6.3	8.0	6.9
	Film B	5.8	4.8	36

[a]Sol = deoxygenated toluene solution (10^{-5} M); Film A = Cz-PAH:mCPBC (1:99, w/w) binary doped film; Film B = Cz-PAH:p4TCzPhBN:mCPBC (1:20:79, w/w/w) ternary doped film. [b] Rate constant of fluorescence radiative decay ($S_1 \rightarrow S_0$, $k_r = \Phi_p/\tau_p$). [c]Rate constant of intersystem crossing ($S_1 \rightarrow T_1$, $k_{ISC} = (1 - \Phi_p)/\tau_p$). [d]Rate constant of reverse intersystem crossing ($T_1 \rightarrow S_1$, $k_{RISC} = \Phi_d/(k_{ISC}\cdot\tau_p\cdot\tau_d\cdot\Phi_p)$).

The Förster radius (R_0) was estimated based on Förster resonance energy transfer (FRET) theory using the spectral overlap between the emission spectrum of the sensitizer (donor) and the absorption spectrum of the terminal dopant (acceptor). The spectral overlap integral (J) was calculated from the normalized donor emission spectrum and the acceptor absorption spectrum. The Förster radius was then calculated according to:

$$R_0^6 = \frac{9(ln10)\kappa^2 \Phi_{\mathrm{D}} J}{128\pi^5 n^4 N_{\mathrm{A}}}$$

where κ^2 is the orientation factor, Φ_{D} is the photoluminescence quantum yield of the donor, n is the refractive index of the medium, and N_{A} is Avogadro's constant. An isotropic orientation of the donor and acceptor molecules was assumed (κ^2 = 2/3). The average donor–acceptor distance (R_{DA}) was estimated according to:

$$R_{\mathrm{DA}} = \left(\frac{3}{4\pi N_{\mathrm{A}} C_{\mathrm{A}}}\right)^{1/3}$$

where C_{A} is the molar concentration of the acceptor in the emitting layer, calculated from the molecular weight, density, and doping concentration. The FRET rate constant (k_{FRET}) was subsequently calculated from R_0, R_{DA}, and the donor lifetime (τ_{D}), according to:

$$k_{\mathrm{FRET}} = \frac{1}{\tau_D}\left(\frac{R_0}{R_{\mathrm{DA}}}\right)^6$$

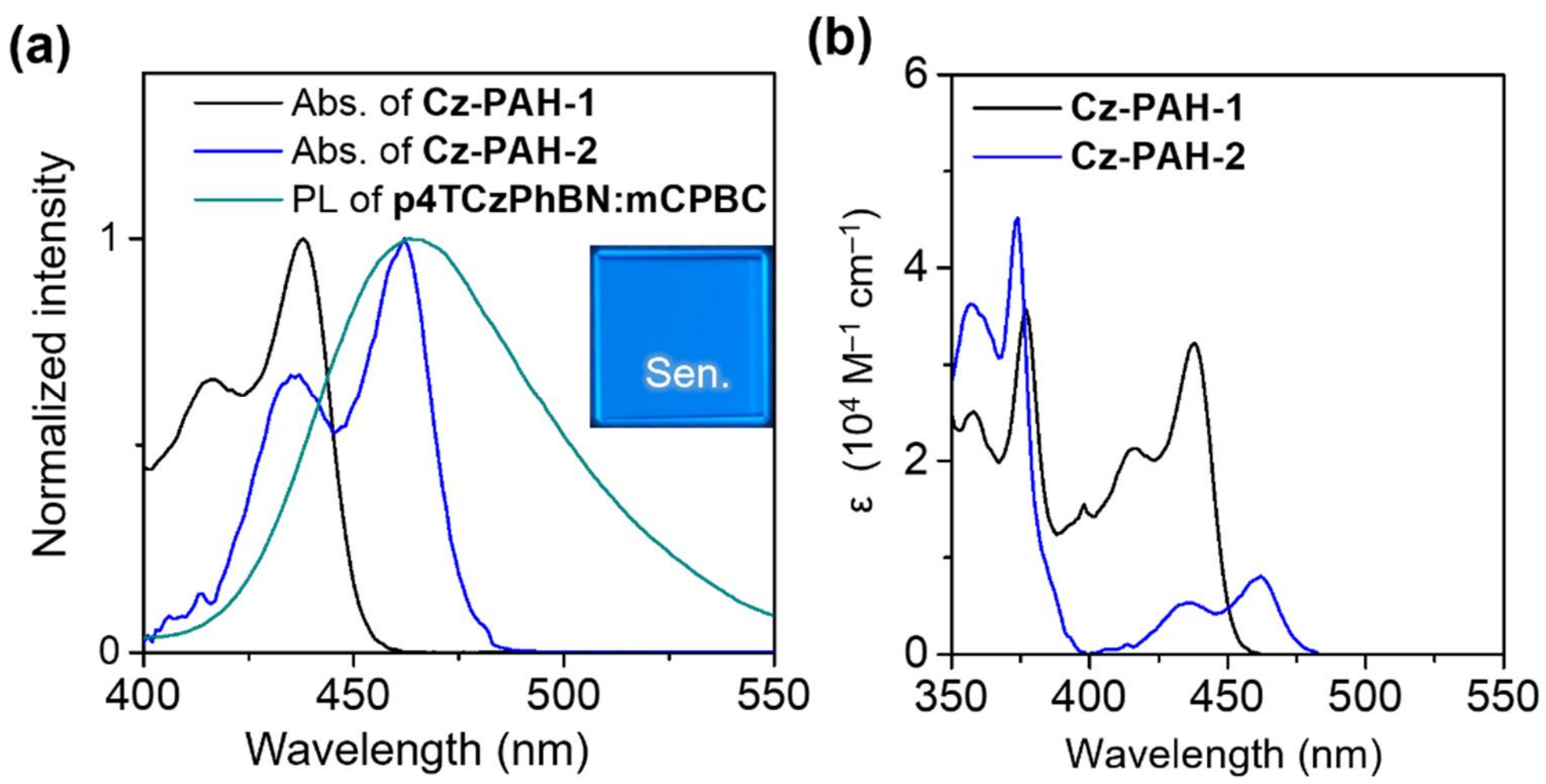

Figure S9. (a) Spectral overlap between the PL spectrum of **p4TCzPhBN** (20 wt% doped in mCPBC) and the absorption spectra of **Cz-PAH-1** and **Cz-PAH-2**. (b) UV–vis absorption spectra of **Cz-PAH-1** and **Cz-PAH-2** in toluene used for the FRET analysis.

Table S6. Calculated FRET parameters for the p4TCzPhBN-sensitized systems using **Cz-PAH-1** and **Cz-PAH-2** as terminal dopants.

	**Cz-PAH-1**	**Cz-PAH-2**
J (M^{-1} cm^{-1} nm^4)	1.66×10^{14}	1.22×10^{14}
R_0 (nm)	3.17	3.01
R_{DA} (nm)	2.44	2.44
k_{FRET} (s^{-1})	1.20×10^{9}	8.81×10^{8}

J: spectral overlap integral; R_0: Förster radius; R_{DA}: average donor–acceptor distance; k_{FRET}: FRET rate constant.

7. OLED fabrication and evaluation

Indium-tin-oxide (ITO)-coated glass substrates were cleaned with detergent, deionized water, acetone, and isopropanol in this order. The substrates were then treated with UV-ozone for 30 min before loading into an E-200 vacuum evaporation system (ALS Technology). Organic layers and a cathode aluminium layer were thermally evaporated on the substrates under vacuum ($< 9 \times 10^{-5}$ Pa) with a deposition rate below 0.5 nm s^{-1} through a shadow mask, defining a pixel size of 0.04 cm^2. The layer thickness and deposition rate were monitored *in situ* using oscillating quartz thickness monitors. The *J*–*V*–*L* characteristics of the fabricated OLEDs were measured using a 2400 source meter (Keithley) and a CS-2000 spectroradiometer (Konica Minolta).

8. Atomic coordinates of optimized Cz-PAH-1 and Cz-PAH-2 (Å)

Cz-PAH-1

C	10.61241269	-2.21463944	-0.39429167
C	9.97296214	-3.45809569	-0.49788775
C	8.58749381	-3.54876883	-0.4690809
C	7.8351858	-2.37966337	-0.3417128
C	8.49788411	-1.13663925	-0.25598229
C	9.8860405	-1.03607206	-0.26875563
N	7.54638247	-0.1254813	-0.13787492
C	6.2850843	-0.69899138	-0.13499601
C	6.42140167	-2.10459035	-0.25470178
C	5.29806212	-2.93719818	-0.26768334
C	4.02757958	-2.39338021	-0.16009522
C	3.90533021	-1.00523394	-0.07370521
C	5.01993828	-0.10060958	-0.08105663
C	4.48789222	1.26446674	-0.05848985
C	4.84152241	2.6196128	-0.13168488
C	3.81922697	3.58649699	-0.12649349
C	2.44480244	3.29593696	-0.07126153
C	2.05815736	1.95426357	-0.0152971
C	0.88217872	1.08867349	-0.00772399
C	-0.48429839	1.35906666	-0.00557945
C	-1.34693113	0.27417067	-0.00779867
C	-0.88217943	-1.08867368	-0.00772575
C	0.48429771	-1.35906671	-0.00558061
C	1.34693066	-0.27417081	-0.00779784
N	2.74412883	-0.24018746	-0.0012084
C	3.11758128	1.06417176	-0.0028203
C	-2.05815825	-1.95426362	-0.01529967
C	-2.44480422	-3.29593664	-0.0712621
C	-3.81922871	-3.58649627	-0.12649472
C	-4.84152395	-2.61961196	-0.13168627
C	-4.48789278	-1.26446633	-0.05849152
C	-5.01993872	0.10061024	-0.08105771
C	-6.28508409	0.69899254	-0.13499789
N	-7.54638363	0.12548241	-0.13786981

C	-8.49788474	1.1366411	-0.25597803
C	-9.88604119	1.03607298	-0.26874831
C	-10.61241369	2.21464026	-0.39428652
C	-9.97296325	3.458096	-0.49788798
C	-8.58749466	3.5487696	-0.46908471
C	-7.83518649	2.37966463	-0.34171403
C	-6.42140162	2.10459102	-0.25470685
C	-5.29806223	2.93719884	-0.2676876
C	-4.02757978	2.39338062	-0.1600979
C	-3.9053302	1.00523453	-0.07370635
N	-2.74412921	0.2401876	-0.00121244
C	-3.11758189	-1.06417155	-0.00282419
H	11.69785405	-2.1683943	-0.41077117
H	10.57003925	-4.36006581	-0.5978752
H	8.09475781	-4.51507419	-0.53969227
H	10.37972521	-0.07301412	-0.18076014
H	5.42244062	-4.01269686	-0.35739829
H	3.14771624	-3.0273256	-0.16182505
H	5.8721687	2.94678022	-0.20701232
H	4.1168173	4.63027533	-0.18162034
H	1.72184012	4.10683176	-0.08839106
H	-0.85118925	2.37997489	-0.00157696
H	0.85118839	-2.37997499	-0.00157819
H	-1.72184237	-4.10683181	-0.0883912
H	-4.11681889	-4.63027464	-0.18162133
H	-5.8721706	-2.94677844	-0.20701259
H	-10.37972484	0.0730147	-0.18074969
H	-11.69785511	2.16839522	-0.41076378
H	-10.57004046	4.36006585	-0.59787714
H	-8.09475913	4.51507475	-0.53970102
H	-5.42243983	4.01269741	-0.35740472
H	-3.14771674	3.02732647	-0.1618275
C	-7.85599855	-1.20462737	0.23414062
C	-8.50868805	-2.04458928	-0.66759962
C	-7.52129211	-1.66196927	1.5090531
C	-8.82054891	-3.3482178	-0.29189241
H	-8.75027666	-1.67178124	-1.65866462

C	-7.82525537	-2.96914568	1.87344648
H	-7.01405644	-0.99140072	2.1961599
C	-8.47555701	-3.81333427	0.97563991
H	-9.32421565	-4.00484914	-0.99576422
H	-7.55333935	-3.32852355	2.8617509
H	-8.71242768	-4.8339024	1.26292886
C	7.85599871	1.20462757	0.23413684
C	7.52128973	1.66196702	1.50904937
C	8.50869319	2.04458901	-0.6675997
C	7.82525521	2.96914155	1.87344717
H	7.01405015	0.99139792	2.19615263
C	8.82055637	3.34821575	-0.29188788
H	8.75028343	1.67178329	-1.65866502
C	8.47556182	3.81333034	0.97564448
H	7.55333697	3.3285177	2.86175154
H	9.32422676	4.00484786	-0.99575644
H	8.71243551	4.83389721	1.26293572

Cz-PAH-2

C	-9.16193197	-3.88422806	0.04744492
C	-7.87591953	-4.44461518	0.05631768
C	-6.75196434	-3.63101379	0.04405947
C	-6.91804856	-2.24467919	0.03178137
C	-8.22156735	-1.69671128	0.03896387
C	-9.35517135	-2.50869248	0.03574512
N	-8.12912771	-0.31142081	0.00983983
C	-6.78237991	0.04569541	-0.01520519
C	-6.21927985	1.31900141	-0.01040671
C	-4.83219345	1.39895727	-0.01650854
C	-3.90944614	2.53258459	-0.01848414
C	-3.862331	3.92963351	-0.01497137
C	-2.6025442	4.55889209	-0.01729143
C	-1.37352901	3.87458385	-0.02207965
C	-1.38964598	2.4754212	-0.02555729
C	-0.52104418	1.30186476	-0.02726907
C	0.86434055	1.15599332	-0.02766556
C	1.36871803	-0.13519595	-0.02708002

C	0.52104401	-1.30186464	-0.02726901
C	-0.86434071	-1.15599316	-0.02766587
C	-1.36871818	0.13519609	-0.02708038
N	-2.69237857	0.57717583	-0.02608536
C	-4.03661597	0.19274648	-0.01905569
C	-4.60164267	-1.06819914	-0.00663043
C	-5.99740859	-1.1363984	-0.00193236
C	-2.66242873	1.93365283	-0.02462
C	1.38964584	-2.4754211	-0.02555713
C	1.37352891	-3.87458374	-0.02207983
C	2.60254407	-4.55889196	-0.01729155
C	3.86233084	-3.92963341	-0.0149712
C	3.90944604	-2.5325845	-0.01848363
C	4.8321934	-1.39895723	-0.01650793
C	6.21927974	-1.3190014	-0.01040622
C	6.78237991	-0.04569544	-0.01520485
N	8.12912771	0.31142071	0.00983982
C	8.22156745	1.69671118	0.03896371
C	9.35517146	2.5086923	0.03574456
C	9.16193224	3.88422784	0.04744435
C	7.87591991	4.44461507	0.05631745
C	6.75196465	3.6310138	0.0440596
C	6.91804872	2.24467915	0.03178152
C	5.9974087	1.13639843	-0.00193194
C	4.6016427	1.06819925	-0.00662982
C	4.03661596	-0.19274642	-0.01905502
N	2.69237847	-0.57717568	-0.02608465
C	2.66242861	-1.93365274	-0.02461944
H	-10.02931121	-4.53874883	0.04634021
H	-7.76182866	-5.52468697	0.067669
H	-5.75495484	-4.0641195	0.03959128
H	-10.35244898	-2.08072672	0.01688499
H	-6.83449227	2.21287625	0.00543931
H	-4.76307801	4.53727097	-0.00923436
H	-2.57836825	5.64528005	-0.0141518
H	-0.44641887	4.44120003	-0.02223526
H	1.51785162	2.02220825	-0.02803421

H	-1.51785173	-2.0222081	-0.02803476
H	-3.99491022	-1.96790259	0.00580905
H	0.44641875	-4.44119989	-0.02223576
H	2.57836811	-5.6452799	-0.0141522
H	4.76307785	-4.53727083	-0.00923426
H	6.83449212	-2.21287625	0.00543975
H	10.35244904	2.08072643	0.01688415
H	10.02931152	4.53874852	0.04633934
H	7.76182903	5.52468692	0.06766874
H	5.75495515	4.06411963	0.03959165
H	3.99491022	1.96790272	0.00580968
C	9.21682956	-0.58725127	0.00611977
C	10.19565298	-0.50270715	0.99871445
C	9.31561086	-1.56192169	-0.98941581
C	11.27204568	-1.38332792	0.98571632
H	10.09744103	0.24683908	1.77848576
C	10.38394979	-2.45240939	-0.98208915
H	8.55881548	-1.60511067	-1.76724252
C	11.36723318	-2.36335938	0.0005112
H	12.03175651	-1.31177311	1.75918587
H	10.45491251	-3.20980182	-1.75789508
H	12.20466866	-3.05522696	-0.00157671
C	-9.21682961	0.58725117	0.00611987
C	-10.19565161	0.50270865	0.99871609
C	-9.31561223	1.56191997	-0.98941716
C	-11.27204425	1.38332947	0.98571813
H	-10.09743854	-0.24683634	1.77848843
C	-10.38395122	2.45240763	-0.98209049
H	-8.55881791	1.60510777	-1.76724492
C	-11.36723323	2.36335928	0.00051146
H	-12.03175397	1.31177596	1.75918887
H	-10.4549151	3.2097988	-1.75789757
H	-12.20466866	3.05522685	-0.00157639

9. References

[1] I. Hattori, M. Hagai, M. Ito, M. Sakai, H. Narita, K. J. Fujimoto, T. Yanai, S. Yamaguchi, *Angew. Chem. Int. Ed.* **2024**, *63*, e202403829.

[2] S. Grimme, C. Bannwarth, P. Shushkov, *J. Chem. Theory Coumput.* **2017**, *13*, 1989-2009.

[3] C. Adamo, V. Barone, *J. Chem. Phys.* **1999**, *110*, 6158-6170.

[4] E. Runge, E. K. Gross, *Phys. Rev. Lett.* **1984**, *52*, 997.

[5] A. Hellweg, S. A. Grüna, C. Hättig, *Phys. Chem. Chem. Phys.* **2008**, *10*, 4119-4127.

[6] M. J. Frisch, G. W. Trucks, H. B. Schlegel, G. E. Scuseria, M. A. Robb, J. R. Cheeseman, G. Scalmani, V. Barone, G. A. Petersson, H. Nakatsuji, X. Li, M. Caricato, A. V. Marenich, J. Bloino, B. G. Janesko, R. Gomperts, B. Mennucci, H. P. Hratchian, J. V. Ortiz, A. F. Izmaylov, J. L. Sonnenberg, D. Williams-Young, F. Ding, F. Lipparini, F. Egidi, J. Goings, B. Peng, A. Petrone, T. Henderson, D. Ranasinghe, V. G. Zakrzewski, J. Gao, N. Rega, G. Zheng, W. Liang, M. Hada, M. Ehara, K. Toyota, R. Fukuda, J. Hasegawa, M. Ishida, T. Nakajima, Y. Honda, O. Kitao, H. Nakai, T. Vreven, K. Throssell, J. J. A. Montgomery, J. E. Peralta, F. Ogliaro, M. J. Bearpark, J. J. Heyd, E. N. Brothers, K. N. Kudin, V. N. Staroverov, T. A. Keith, R. Kobayashi, J. Normand, K. Raghavachari, A. P. Rendell, J. C. Burant, S. S. Iyengar, J. Tomasi, M. Cossi, J. M. Millam, M. Klene, C. Adamo, R. Cammi, J. W. Ochterski, R. L. Martin, K. Morokuma, O. Farkas, J. B. Foresman, D. J. Fox, Rev. C.01 ed., Gaussian, Inc., Wallingford, CT, **2019**.

[7] Y. J. Franzke, C. Holzer, J. H. Andersen, T. Begusic, F. Bruder, S. Coriani, F. Della Sala, E. Fabiano, D. A. Fedotov, S. Furst, S. Gillhuber, R. Grotjahn, M. Kaupp, M. Kehry, M. Krstic, F. Mack, S. Majumdar, B. D. Nguyen, S. M. Parker, F. Pauly, A. Pausch, E. Perlt, G. S. Phun, A. Rajabi, D. Rappoport, B. Samal, T. Schrader, M. Sharma, E. Tapavicza, R. S. Tress, V. Voora, A. Wodynski, J. M. Yu, B. Zerulla, F. Furche, C. Hattig, M. Sierka, D. P. Tew, F. Weigend, *J. Chem. Theory Comput.* **2023**, *19*, 6859-6890.

[8] M. Hagai, N. Inai, T. Yasuda, K. J. Fujimoto, T. Yanai, *Sci. Adv.* **2024**, *10*, eadk3219.

[9] N. Inai, S. Yamaguchi, T. Yanai, *ACS Phys. Chem Au* **2023**, *3*, 540-552.

[10] Y. Niu, Q. Peng, C. Deng, X. Gao, Z. Shuai, *J. Phys. Chem. A* **2010**, *114*, 7817-7831.

[11] C. Chen, S. P. Ong, *Nat. Comput. Sci.* **2022**, *2*, 718-728.

[12] O. Vinyals, S. Bengio, M. Kudlur, *International Conference on Learning Representations (ICLR)* **2016**, arXiv:1511.06391.

[13] H. L. Lee, S. O. Jeon, I. Kim, S. C. Kim, J. Lim, J. Kim, S. Park, J. Chwae, W. J. Son, H. Choi, J. Y. Lee, *Adv. Mater.* **2022**, *34*, e2202464.

[14] K. Goushi, K. Yoshida, K. Sato, C. Adachi, *Nat. Photon.* **2012**, *6*, 253-258.
